# Identification of an inflammation-related risk signature for prognosis and immunotherapeutic response prediction in bladder cancer

**DOI:** 10.1038/s41598-024-51158-9

**Published:** 2024-01-12

**Authors:** Yanjun Wang, Yi Tang, Zhicheng Liu, Xingliang Tan, Yuantao Zou, Sihao Luo, Kai Yao

**Affiliations:** 1 Department of Urology, Sun Yat-Sen University Cancer Center, Guangzhou, 510060 China; 2 State Key Laboratory of Oncology in Southern China, Guangzhou, 510060 China; 3 Collaborative Innovation Center of Cancer Medicine, Guangzhou, 510060 China; 4 Guangdong Provincial Clinical Research Center for Cancer, Guangzhou, 510060 China

**Keywords:** Bladder cancer, Cancer genomics

## Abstract

Tumor inflammation is one of the hallmarks of tumors and is closely related to tumor occurrence and development, providing individualized prognostic prediction. However, few studies have evaluated the relationship between inflammation and the prognosis of bladder urothelial carcinoma (BLCA) patients. Therefore, we constructed a novel inflammation-related prognostic model that included six inflammation-related genes (IRGs) that can precisely predict the survival outcomes of BLCA patients. RNA-seq expression and corresponding clinical data from BLCA patients were downloaded from The Cancer Genome Atlas database. Enrichment analysis was subsequently performed to determine the enrichment of GO terms and KEGG pathways. K‒M analysis was used to compare overall survival (OS). Cox regression and LASSO regression were used to identify prognostic factors and construct the model. Finally, this prognostic model was used to evaluate cell infiltration in the BLCA tumor microenvironment and analyze the effect of immunotherapy in high- and low-risk patients. We established an IRG signature-based prognostic model with 6 IRGs (TNFRSF12A, NR1H3, ITIH4, IL1R1, ELN and CYP26B1), among which TNFRSF12A, IL1R1, ELN and CYP26B1 were unfavorable prognostic factors and NR1H3 and ITIH4 were protective indicators. High-risk score patients in the prognostic model had significantly poorer OS. Additionally, high-risk score patients were associated with an inhibitory immune tumor microenvironment and poor immunotherapy response. We also found a correlation between IRS-related genes and bladder cancer chemotherapy drugs in the drug sensitivity data. The IRG signature-based prognostic model we constructed can predict the prognosis of BLCA patients, providing additional information for individualized prognostic judgment and treatment selection.

## Introduction

Bladder urothelial carcinoma (BLCA) is the second most common urological malignancy, with 91,893 and 84,825 new cases per year in China and the United States, respectively, and poses a fatal threat to human health, with an estimated 42,973 and 19,223 deaths in 2022, respectively^1,2^. The majority of patients are diagnosed initially with nonmuscle invasive BLCA with a favorable prognosis, but progression and metastasis occur in 30% of patients with poor outcomes due to the complex and unclear mechanisms involved in the development of BLCA^2^. The TNM staging system, pathological differentiation degree and molecular stratification have been widely used for detecting high-risk BLCA patients but are still insufficient for precise and individualized prognostic prediction. Therefore, more attention should be focused on identifying effective biomarkers to forecast the clinical outcomes of BLCA for early management and reduce the additional therapeutic burden on patients.

Inflammation is one of the ten characteristics of tumors^3^. Tumor-associated inflammation helps incipient neoplasia to acquire hallmark capabilities and is closely related to tumor occurrence and development^3^. Substantial evidence has suggested that high-risk factors such as cigarette smoking, exposure to aromatic amines, schistosome infections and endogenous irritants can induce chronic and persistent bladder inflammation, which plays a direct etiological role in carcinogenesis and promotes the progression of BLCA^4–6^. In addition, tumor cells secrete inflammatory molecules connected with immune and stromal cells, including cytokines, to shape the inflammatory tumor microenvironment; growth factors, which sustain proliferative signals and prevent cell death; and extracellular matrix-modifying enzymes, which activate invasion and metastasis^7–9^. Recent immunotherapies have also been used in bladder cancer treatment. The tumor inflammatory environment is associated with an inhibitory immune microenvironment, which has been found in previous studies to modulate PDL1 expression and influence the efficacy of immunotherapy in patients with bladder cancer^10,11^. However, tumor inflammation is a dynamic process associated with the expression of multiple genes associated with high tumor heterogeneity and has not been investigated in BLCA.

In this study, we constructed a novel inflammation-related prognostic model comprising six inflammation-related genes (IRGs), TNFRSF12A, NR1H3, ITIH4, IL1R1, ELN and CYP26B1, by univariate and LASSO Cox regression analyses of data from The Cancer Genome Atlas (TCGA) database. The model was further validated with the GSE32894 dataset to determine its stability and reliability and was regarded as an independent indicator of survival in BLCA patients. In addition, somatic mutation information was obtained, a nomogram was constructed, clinical characteristic stratification was performed, and the tumor microenvironment (TME) landscape and chemotherapeutic response prediction were performed based on the risk of inflammation-related prognosis. Finally, the mRNA expression of IRGs was detected in BLCA cell lines and normal urothelial epithelial controls.

## Methods

### Data source and preprocessing

The RNA-seq expression data of BLCA patients, corresponding clinical characteristics and nucleotide variation data were downloaded from the TCGA database^12^. The TCGA-BLCA cohort containing 411 tumor and 19 normal tissue samples was used to construct the prognostic signature of the IRGs. The GSE32894 dataset, which included 308 tumor samples, was used as the validation cohort^13^. In addition, in the cohort of patients, a urothelial carcinoma cohort treated with atezolizumab was used to predict the response to immunotherapy^14^. For the above datasets, RNA-seq data (FPKM values) were log_2_ (FPKM + 1) normalized. The panel of IRGs was combined with inflammation-associated genes from the NCBI-Gene website and a published panel.

### Differentially expressed IRGs screening and gene mutation analysis

Differentially expressed IRGs were detected between normal and tumor tissues in the TCGA-BLCA cohort via the R package “DESeq2 v1.32.0”^15^. The cutoff values were regarded as |logfoldchange (FC)|> 1.0 and a false discovery rate (FDR) < 0.05. The differentially expressed IRGs were subsequently subjected to gene ontology (GO) and Kyoto encyclopedia of genes and genomes (KEGG) enrichment analyses via the R package “clusterProfiler v4.0.5”. The somatic mutations of the IRGs were analyzed by the R package “maftools v2.8.05” and are shown as a landscape heatmap.

### Gene set enrichment analysis (GSEA)

GSEA was conducted on the RNA-seq data of 486 differentially expressed IRGs by GSEA tools version 4.1 (http://www.broadinstitute.org/gsea). We analyzed the subsets of the Molecular Signatures Database (C2 and C5) as previously described and calculated the corresponding *p* values.

### Construction and validation of the IRG prognostic model

Univariate Cox regression was performed to identify overall survival-related differentially expressed IRGs. Survival analysis was performed with Kaplan–Meier survival curves, and the mean expression level of each gene was used as the cutoff. Survival-related differentially expressed IRGs were ranked according to hazard ratio (HR) and displayed in a forest plot. Subsequently, LASSO regression analyses were performed with the R package “glmnet v 4.1.1” to remove redundant factors and construct an optimal IRG signature-based prognostic model to evaluate the survival of BLCA patients. The risk score of the IRG prognostic regression model was multiplied by the expression level of the six IRGs and the corresponding coefficient, as previously described. The mean risk score was the cutoff value for distinguishing between the high- and low-risk groups and was validated in the GSE32894 cohort. The predictive value of the R package “survivalROC v1.0.3” was detected by the area under the curve (AUC). In addition, we performed correlation analysis between risk scores and clinical features using stratification analysis and nomograms. The consistency between the predicted 1-, 3-, and 5-year survival probabilities and the actual survival probabilities was evaluated using calibration plots.

### Estimation of cell infiltration in the tumor microenvironment (TME) and the clinical significance of the IRG prognostic model

A prognostic model of six IRGs was used to evaluate cell infiltration in the BLCA TME. The immune landscape was explored by using CibersortABS and the R package “xCell v1.1.0”. Single-sample GSEA (ssGSEA) was used to calculate the difference in the cell composition in the TME between the high- and low-risk groups. To analyze the difference in immunotherapy efficacy between the high- and low-risk groups, we downloaded the IMvigor210 immunotherapy cohort and conducted Kaplan–Meier survival analysis and AUC prediction. The relationship between the IRG risk score and clinical characteristics was detected in the TCGA-BLCA cohort. A significant difference was regarded as *p* < 0.05 according to one-way analysis of variance (ANOVA).

### Chemotherapy response analysis

The RNA-seq data of NCI-60 cancer cell lines were downloaded from the CellMiner database (https://discover.nci.nih.gov/cellminer) published by the National Institute of Health. The relationships of the expression of the six target IRGs between BLCA cell lines and 411 chemotherapy drugs that passed clinical trials with FDA approval were explored by Pearson correlation analysis. A *p* value < 0.05 was considered to indicate drug susceptibility.

### Cell lines and culture conditions

Immortalized human normal urothelial epithelial SV-HUC-1 cells were purchased from American Type Culture Collection. Urothelial carcinoma cell lines, including T24, 5637, RT4, BIU-87 and UM-UC-3 cells, were obtained from Professor Kai Yao. SV-HUC-1 cells were cultured in Ham's F-12K (Kaighn's) medium; T24 and 5637 cells were cultured in RPMI 1640 medium; and RT4, BIU-87 and UM-UC-3 cells were cultured in Dulbecco's modified Eagle’s medium. All of the media were supplemented with 10% fetal bovine serum and 1% penicillin/streptomycin and incubated at 37 °C in 5% carbon dioxide.

### Real-time-quantitative polymerase chain reaction (qPCR)

The mRNA expression of six IRGs was detected in BLCA cell lines and SV-HUC-1 cells. Total RNA extraction (RC101, Vazyme), reverse transcription (R122-01, Vazyme) and cDNA amplification (Q711-02/03, Vazyme) were performed according to protocols described previously. Using the 2(−∆∆Ct) method, relative target gene expression was quantified and normalized against that of GAPDH. The sequences of primers used are listed in Table S5.

### Ethics approval and consent to participate

This study was approved by the Ethics Committee of SYSUCC (SZR2022-001).

## Results

### Identification of differentially expressed IRGs and somatic mutation analysis

**Figure 1:**
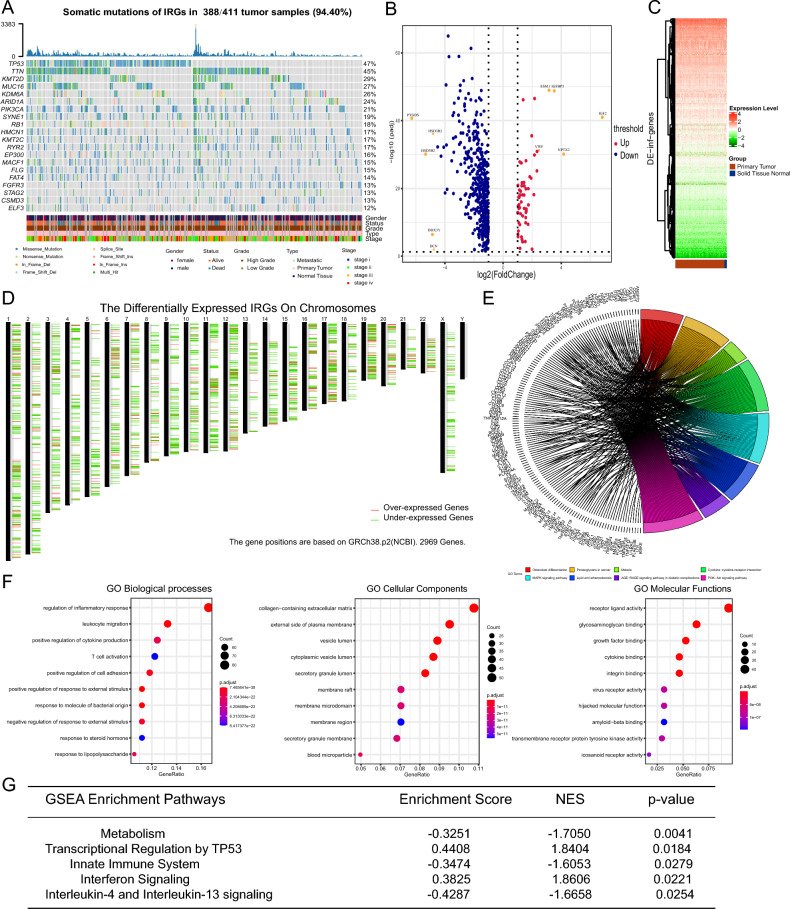
Identification of differentially expressed IRGs in BLCA patients. (**A**) Among 411 patients, TP53, TTN, KMT2D, MUC16 and KDM6A were found to be the 5 most frequently mutated genes. (**B**–**D**) Among the differentially expressed IRGs between tumor tissues and normal tissues, 59 were upregulated and 426 were downregulated according to volcano plots, heatmaps and chromosome schematics. (**E**–**F**) The GO, KEGG and GSEA results showed that the IRGs in BLCA were enriched mainly in the activation of the inflammatory response, leukocyte secretion and regulation of tumor immunity. (**G**) GSEA showed that the differentially expressed IRGs were enriched in the regulation of interferon and the innate immune system.

Combined with the NVCI-Gene database and a previously reported cluster of inflammation-associated genes, a total of 2343 IRGs were included in this study and are listed in Table S1. Differentially expressed IRGs were screened between 411 BLCA tumors and 19 normal tissues from the TCGA database. The detailed clinical information is listed in Table S2. Genomic mutation analysis indicated that most of the BLCA patients (388/411, 94.4%) had at least one somatic mutation in an IRG, suggesting the predisposing role of IRG mutations in BLCA (Fig. 1A). The mutation frequencies of TP53 (47%), TTN (45%), KMT2D (29%), MUC16 (27%) and KDM6A were the 5 most common IRGs in BLCA (Fig. 1A). Subsequently, the differentially expressed IRGs between the tumors and normal tissues were detected, and the results revealed a total of 59 significantly upregulated and 426 downregulated IRGs (Fig. 1B and Table S3). The differential IRGs were visualized by volcano plot and chromosome schematic (Fig. 1C,D).

### Biological function analysis of differentially expressed IRGs

A total of 485 differentially expressed IRGs were obtained and subjected to GO, KEGG and GSEA to explore the enrichment of biofunctions. The results indicated that the IRGs in BLCA were enriched mainly in the activation of the inflammatory response, leukocyte secretion and regulation of tumor immunity (Fig. 1E,F). GSEA revealed that the differentially expressed IRGs were enriched in the regulation of interferon and the innate immune system (Fig. 1G). The biological functional results suggested that IRG signatures promoted an inflammatory microenvironment in BCLA patients, which might be essential for tumor progression and associated with clinical outcomes.

### Generation of IRG prognostic signatures in BLCA

**Figure 2:**
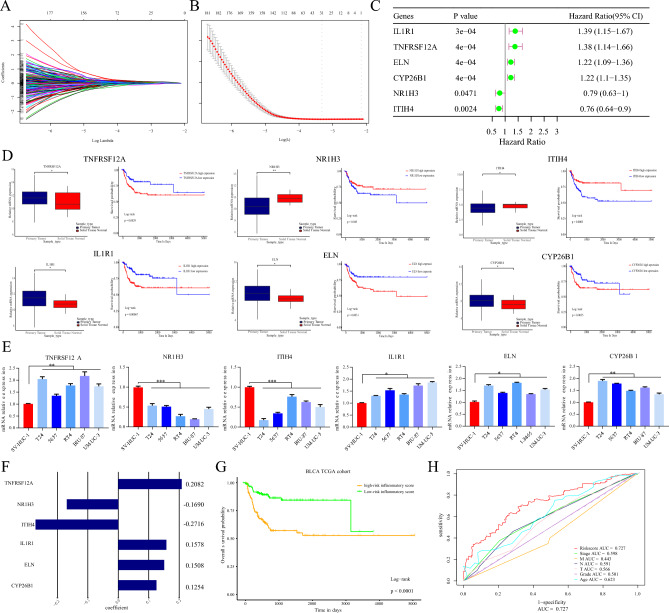
The prognostic value of differentially expressed IRGs in BLCA. (**A**,**B**) The LASSO Cox regression model was used to construct a risk stratification model of the prognostic signatures of the 6 differentially expressed IRGs. (**C**) Hazard ratios of the IRGs are presented with forest plots. (**D**) Among the 6 IRGs, TNFRSF12A, IL1R1, ELN and CYP26B1 were unfavorable prognostic factors, and NR1H3 and ITIH4 were protective factors; (**E**) TNFRSF12A, IL1R1, ELN and CYP26B1 were overexpressed in bladder cancer cell lines, while NR1H3 and ITIH4 were downregulated. (**F**) The coefficients of the six genes related to the inflammatory response that were screened by LASSO regression. (**G**) Survival analysis revealed that high-risk patients had significantly poorer OS than low-risk patients. (H) The IRG prognostic signature had a greater AUC than did the traditional clinicopathological factors.

To further determine the prognostic value of the differentially expressed IRGs in BLCA, univariate survival analysis was performed, and the results indicated that 113 IRGs were associated with overall survival (Table S4). To remove confounding factors, a LASSO Cox regression model was used to construct a risk stratification model of six IRG prognostic signatures, namely, TNFRSF12A, NR1H3, ITIH4, IL1R1, ELN and CYP26B1 (Fig. 2A,B). The hazard ratios (HRs) of the six survival-related IRGs are presented as forest plots (Fig. 2C). We detected that TNFRSF12A, IL1R1, ELN and CYP26B1 were unfavorable prognostic factors that were overexpressed in tumor tissues and associated with poor survival in the TCGA-BLCA cohort. However, NR1H3 and ITIH4 are protective indicators that are overexpressed in normal tissues and predict better outcomes (Fig. 2D). Similarly, qPCR was performed to detect the mRNA expression of six target genes in bladder cancer cell lines and the corresponding normal epithelial cell line SV-HUC-1. We found that TNFRSF12A, IL1R1, ELN and CYP26B1 were overexpressed in bladder cancer cell lines, while NR1H3 and ITIH4 were downregulated (Fig. 2E). Subsequently, the risk scores of the IRG prognostic signatures were calculated by the target gene expression and the corresponding coefficients (Fig. 2F). The mean risk score was 3.552 in the TCGA-BLCA cohort and was regarded as the threshold of the IRG prognostic model. Survival analysis indicated that high-risk patients had significantly poorer overall survival (OS) than did patients with lower risk scores (*p* < 0.0001) (Fig. 2G). The area under the curve (AUC) curves showed that the predictive efficiency of the IRG prognostic signature had the highest AUC value (0.727) compared with that of traditional clinicopathological factors (Fig. 2H). In brief, we established an available IRG prognostic model to evaluate the clinical outcomes of BLCA patients.

### The clinical significance of the IRG prognostic model in the TCGA-BLCA cohort

**Figure 3:**
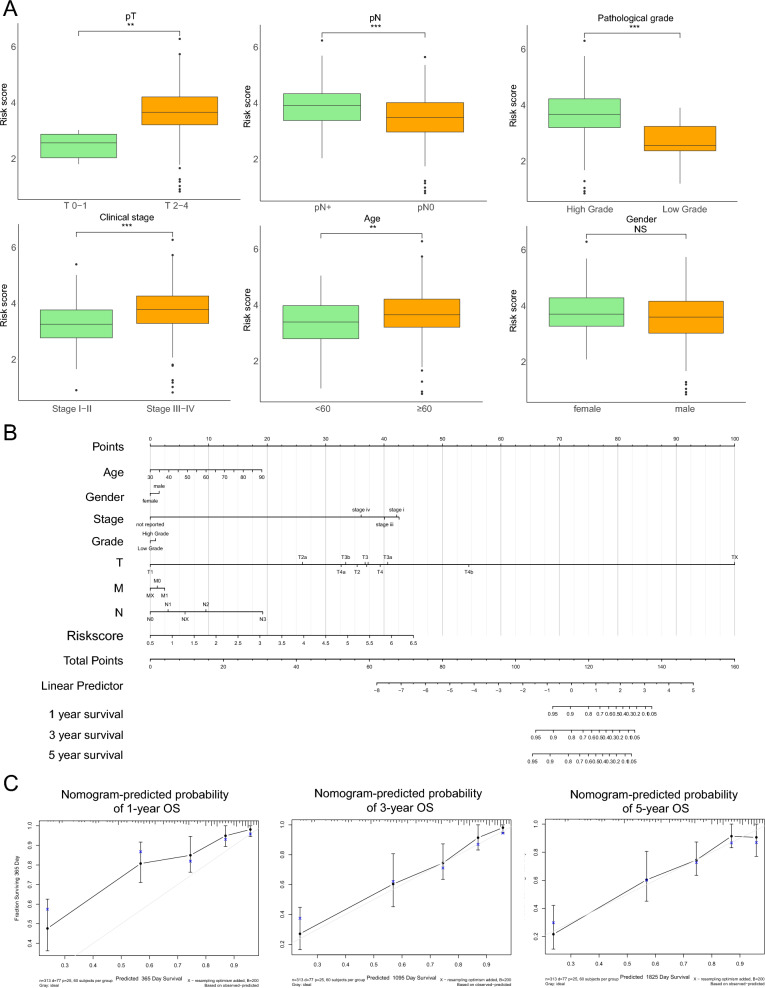
The clinical significance of the IRG prognostic model in the TCGA-BLCA cohort. (**A**) Advanced T stage, lymph node metastasis, pathological grade, poor clinical stage and age ≥ 60 years were positively associated with high-risk IRG scores. (**B**) Survival nomograms were constructed with prognosis-related clinical factors and IRG signatures. (**C**) The nomogram-predicted 1-, 3- and 5-year survival rates were highly consistent with the actual survival rates in the TCGA-BLCA cohort. *p < 0.05; **p < 0.01; ***p < 0.001; ****p < 0.0001. *NS* not significant.

To further demonstrate the clinical significance of the IRG prognostic model in the TCGA-BLCA cohort, patients were divided into two groups based on clinicopathological factors. Correlation analysis indicated that advanced T stage (pT2–pT4), lymph node metastasis, pathological grade, poor clinical stage and age (≥ 60) were positively associated with high-risk IRG score, suggesting poor survival (Fig. 3A). In addition, we combined prognosis-related clinical factors and IRG signatures to construct a survival nomogram (Fig. 3B). Calibration curves from the TCGA-BLCA cohort showed that the nomogram-predicted 1-, 3- and 5-year survival rates were highly consistent with the actual survival rates, which demonstrated that the IRG prognostic signatures were stable and effective (Fig. 3C).

### External validation of the IRG prognostic signatures

**Figure 4:**
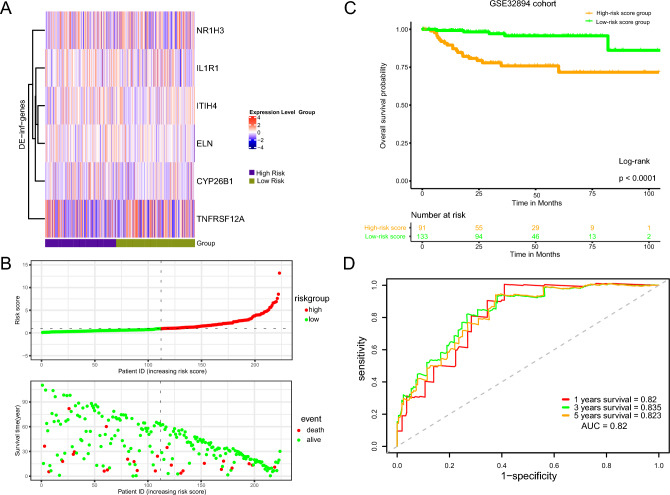
External validation of the IRG prognostic signatures. (**A**,**B**) The IRG signature expression map and the distribution of risk scores of 133 BLCA patients in the GSE32894 cohort are shown. (**C**) Patients in the high-risk score group had poorer OS than patients in the low-risk score group. (**D**) The area under the curve (AUC) of the IRG prognostic model indicated good performance in predicting patient prognosis.

To further explore the efficacy of the IRG prognostic signatures, we used the GEO database (GSE32894) for independent external validation. In the GSE32894 cohort, 133 BLCA patients were divided into a low-risk group with an IRG risk score lower than 0.2, and 91 other patients were included in the high-risk group. The IRG signature expression map and the distribution of risk scores are shown in Fig. 4A,B. Survival analysis demonstrated that the overall survival (OS) of high-risk BLCA patients was significantly shorter than that of low-risk patients (*p* < 0.0001) (Fig. 4C). The area under the curve (AUC) curves of the IRG prognostic model indicated even better performance, with AUC values of 0.82, 0.835 and 0.823 at 1 year, 3 years and 5 years, respectively, in predicting survival (Fig. 4D).

### The predictive value of the IRG prognostic signature for immune cell infiltration and immunotherapy efficacy

**Figure 5:**
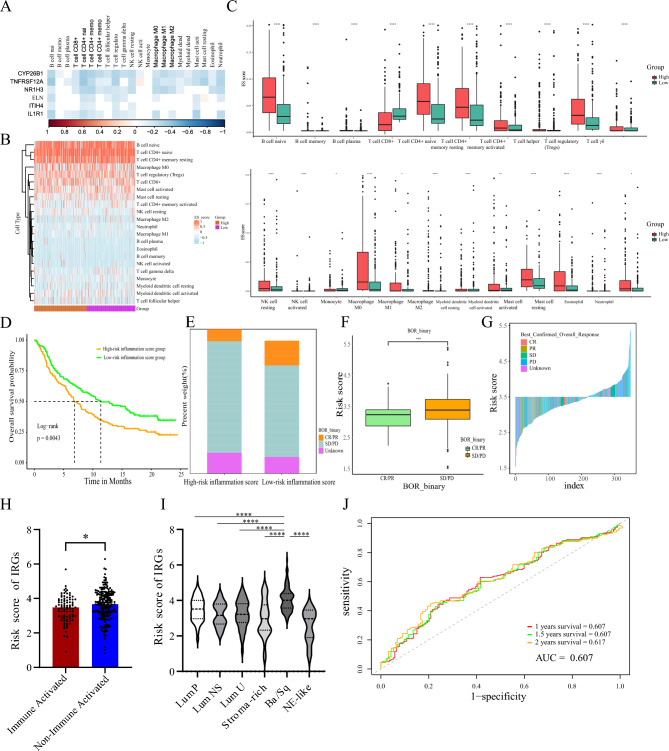
The predictive value of the IRG prognostic signature for immune cell infiltration and immunotherapy efficacy. (**A**,**B**) The high-risk IRG group had abundant immune cell types and proportions. (**C**) The proportions of naive immune cells, Treg cells, M2 TAMs, and myeloid dendritic cells were increased, while the percentage of CD8+ T cells was decreased in the high-risk group. (**D**) Survival analysis of patients in the IMvigor210 cohort showed that the OS of urothelial carcinoma patients with high-risk IRG signatures was significantly shorter than that of patients with low-risk signatures. (**E**) The proportion of patients with objective response rates in the low-risk group was greater than that in the high-risk group. (**F**–**G**) Patients in the database who responded to atezolizumab had lower risk scores. (**H**) Risk scores of IRGs between the immune-activated and nonimmune-activated groups. (**I**) Risk scores of IRGs in six molecular typologies. (**J**) The AUC of the IRG prognostic signature was 0.607.

Biological enrichment analysis revealed that the differentially expressed IRGs were associated with the regulation of tumor immunity. To further understand the relationships between the risk stratification of the IRG prognostic signatures and immune cell infiltration, we evaluated the immune cell landscape in the TCGA-BLCA cohort with the CIBERSORT tool. A heatmap indicated that the high-risk IRG group had abundant immune cell types and proportions, suggesting an inflammation-associated immune microenvironment in BLCA (Fig. 5A,B). However, in the high-risk group, the increase in naive immune cells, Treg cells, M2 TAMs, and myeloid dendritic cells and the decrease in CD8+ T cells constituted a suppressive microenvironment to evade immune surveillance, resulting in poor prognosis (Fig. 5C). Moreover, we further validated the predictive value of the IRG prognostic signature for immunotherapy efficacy in patients in the IMvigor210 cohort. Survival analysis indicated that the overall survival of urothelial carcinoma patients with high-risk IRG signatures was significantly shorter than that of patients with low-risk signatures (Fig. 5D). In the low-risk IRG group, the proportion of patients with objective response rates was greater than that in the high-risk group (11.7% vs. 29.79%, *p* < 0.001) (Fig. 5E). Moreover, patients who responded to atezolizumab in the database had lower risk scores (Fig. 5F,G). After categorizing all patients into immune-activated and nonimmune-activated groups (including both immune exhausted and nonimmune patients) based on Meng et al.^16^, we compared the inflammation scores between the two groups and found that lower inflammatory scores in patients with immune activation status suggested a possible benefit from immunotherapy (Fig. 5H). Among the six molecular subtypes, the basal/squamous subtype exhibited the highest degree of inflammation, which was significantly different from that of the other subtypes. (Fig. 5I) The AUC of the IRG prognostic signature was 0.607 for predicting the clinical outcome of immunotherapy (Fig. 5J). Our results demonstrated that high-risk BLCA patients in the IRG prognostic model were associated with an inhibitory immune tumor microenvironment and poor immunotherapy response.

### Prediction of the chemotherapy response in the signature

**Figure 6:**
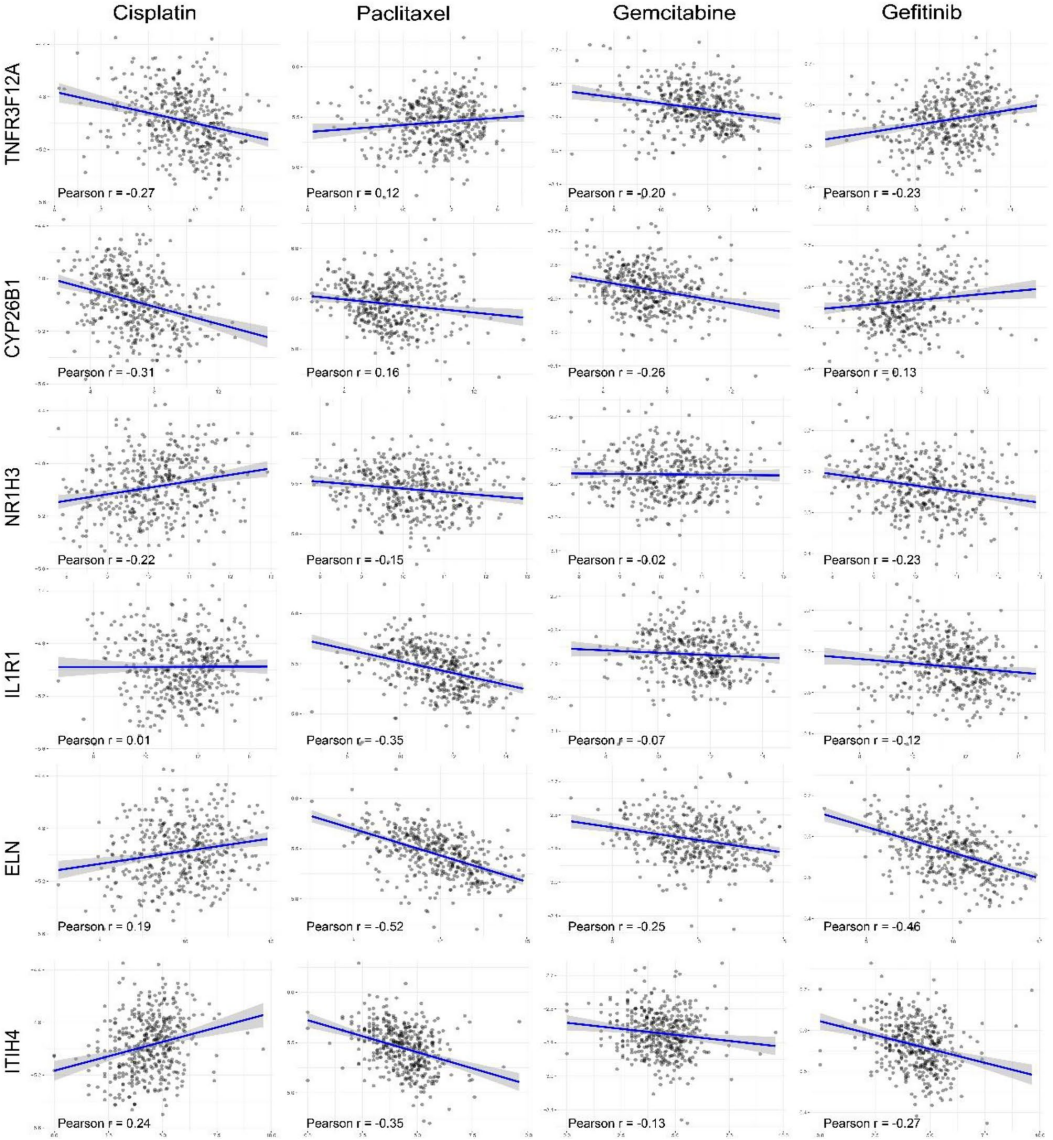
Prediction of chemotherapy response according to the signature. In the high-risk IRG cohort, IL1R1, ELN and ITIH4 overexpression was negatively correlated with the first-line chemotherapy drugs cisplatin and gemcitabine, while the efficacy of paclitaxel was sensitive to the upregulation of ELN and ITIH4 expression.

To investigate the association between chemotherapy outcomes and the expression pattern of the IRG signature, we explored drug sensitivity data from the Cell Miner database. The results suggested a correlation between IRS-related genes and bladder cancer chemotherapy drugs (Fig. 6). In the high-risk IRG cohort, IL1R1, ELN and ITIH4 overexpression was negatively correlated with the first-line chemotherapy drugs cisplatin and gemcitabine, indicating that chemotherapy may not be effective. In contrast, the efficacy of paclitaxel was sensitive to the upregulation of ELN and ITIH4 expression; thus, paclitaxel might serve as an available chemotherapy option (Fig. 6). In brief, the IRG prognostic signatures provide additional information and a reference for individualized chemotherapy in BLCA patients.

## Discussion

Increasing evidence has suggested that tumor-associated inflammation, including gene toxicity, aberrant tissue repair, proliferation, invasion, and metastasis, is causally associated with cancer development^17,18^. Due to the close relationship between inflammation and cancer, the correlation of inflammatory signatures with disease diagnosis or clinical endpoints has been studied in many cancer types^19,20^. Qiu et al*.* explored a four-gene inflammation-related signature that included IL13, BDNF, PLCG2 and TIMP1 and could predict the prognosis and treatment response in patients with colon adenocarcinoma^21^. Zhang et al*.* identified 10 differentially expressed inflammation-related lncRNAs to predict individualized clinical outcomes in gastric carcinoma patients^22^. However, inflammation-induced carcinogenesis is the result of interactions among multiple intrinsic and extrinsic cellular processes, including genomic instability, reprogramming of the stromal environment, cytokine secretion and the immune response, which contribute to the high degree of heterogeneity among different types of tumors^23,24^. BLCA is a chronic inflammation-related cancer and is also referred to as a “hot tumor” due to the increased infiltration of activated immunocytes and inflammatory-related cells^25,26^. However, the potential role of inflammation-related genes in BLCA is unknown, and inflammation-related prognostic signatures for identifying effective immunotherapy strategies are lacking.

Here, we constructed a novel IRG classifier that included TNFRSF12A, NR1H3, ITIH4, IL1R1, the ELN and CYP26B1 and explored its prognostic value for predicting overall survival (OS) and the response to immunotherapy. Among the six IRGs, TNFRSF12A is an aging-related gene involved in the hypoxia-driven inflammatory response and contributes to thyroid cancer^27,28^. A low NR1H3 expression level has been verified to be an independent prognostic factor for poor overall survival and predicts worse recurrence-free survival in muscle-invasive bladder cancer patients^29^. ITIH4 is a serum inflammation biomarker for early gastric cancer diagnosis^30^. The immune-related genes IL1R1 and ELN predict poor survival in patients with pancreatic adenocarcinoma and bladder cancer, respectively^31^. CYP26B1 is required for the activation of T cells via retinoic acid-dependent signals that participate in the immune response^32^. These studies support that the six IRGs included in our classifier are potentially measurable indicators of prognosis in BLCA patients.

To further demonstrate the clinical significance of our six-IRG signature in BLCA, we first divided patients into high-risk and low-risk groups according to the median IRG risk score. Correlation analysis indicated that advanced pT stage, pN stage, pathological grade, and clinical stage were strongly associated with high-risk IRG scores. Moreover, the six-IRG-related signature presented a strong ability to predict overall survival, with an AUC of 0.727 in the TGCA-BLCA cohort and even better performance in the GSE32894 cohort (AUC of 0.820). Compared with traditional prognostic factors such as the TNM staging system and clinical stage, our six-IRG-related signature was more effective and was an independent prognostic factor for BLCA (Fig. 2G). Previous studies have shown that the basal/squamous subtype of bladder cancer is linked to chronic inflammation of the bladder and has a poorer prognosis than other subtypes^33,34^. Our findings further support this association. In recent years, improvements in whole-genome sequencing have facilitated a deeper understanding of genomic alterations in BLCA. Wang et al*.* identified a seven-lncRNA signature with an area under the curve (AUC) of 0.734, which has robust efficacy in predicting overall survival in patients with BLCA^35^. Similarly, Wu et al*.* established an eight-immune-related lncRNA signature for predicting patient prognosis, for which the area under the curve (AUC) values at 1, 3 and 5 years were 0.72, 0.76 and 0.76, respectively^36^. Compared with the above studies, our study incorporated the inflammatory drivers of BLCA to create a more concise inflammation-related prognostic signature with comparable predictive efficacy.

In addition, an active inflammatory reaction recruits tumor-infiltrating lymphocytes into the tumor microenvironment through the release of cytokines, tumor necrosis factors and growth factors and leads to dramatic differences in immunotherapy responses^23^. We found that BLCA patients with high-risk inflammatory scores suffer from low response rates to PD-L1 blockade, which is associated with poor survival. The AUC value of our six-IRG signature was 0.607 for predicting immunotherapy response in the IMvigor210 database, and the effect was similar to that of another nine immune-relevant gene signatures (AUC = 0.64, 95% = 0.55–0.74), which was reported by Jiang et al*.*^37^. Our results also indicated that the proportion of immunosuppressive cells, such as Treg cells and M2 TAMs, was significantly increased in high-risk patients, which is key to the formation of an immunosuppressive microenvironment and tumor immune evasion. M2 TAMs recruit Treg cells by secreting CCL22 and synergistically produce IL-10 and TGF-β to inhibit the activation and proliferation of T cells^38,39^. In addition, the reductions in dendritic cells and CD8+ T cells in high-risk patients inhibited antigen presentation and cytotoxicity, respectively, resulting in a low immunotherapy response and poor survival. In brief, our six-IRG signature was beneficial for identifying the tumor inflammatory microenvironment in BLCA patients and predicting the efficacy of immunotherapy.

Finally, our six-IRG signature also provided evidence for the effectiveness of chemotherapy in guiding personalized treatments. Gemcitabine and cisplatin are commonly used as first-line chemotherapies in combination and have shown clinical benefit in treating BLCA^40,41^. We detected that BLCA patients with high IRG risk scores, especially those with overexpression of ITIH4 and IL1R1, were less sensitive to gemcitabine and cisplatin chemotherapy. However, patients in which the oncogenes TNFR3F12A and CYP26B1 were downregulated had a better response to gemcitabine and cisplatin as well as a better prognosis. On the other hand, paclitaxel has been shown to be an active front-line and palliative therapy in BLCA^42,43^. Alternative regimens, including cisplatin/paclitaxel and gemcitabine/paclitaxel, have shown modest activity in phase I-II trials^44,45^. We found that high TNFR3F12A expression or low NR1H3 and ITIH4 expression was positively correlated with paclitaxel sensitivity, indicating that high-risk BLCA patients could benefit from second-line paclitaxel chemotherapy.

Our study has several limitations. First, the signature has been validated retrospectively in only the GEO database, and future prospective studies are needed to confirm its clinical value. Furthermore, further in vivo and in vitro studies are needed to determine how the six selected genes contribute to the development of BLCA. Despite the limited sample size in this study, we explored an inflammation-related prognostic signature and assessed the response to immunotherapy. This model provides useful information for individualized clinical treatment and prognosis judgment.

## Conclusion

In this study, we constructed an available six-IRG signature based on the TCGA and GEO cohorts to predict the prognosis of BLCA patients. We also examined the gene mutation status, immune landscape and drug sensitivity among the different risk groups. Our inflammation-associated signature provides additional information for individualized prognostic judgment and treatment selection.

### Supplementary Information

Supplementary Tables.

## Data Availability

The datasets supporting the conclusions of this article are available from the corresponding author upon reasonable request. The authenticity of this article has been validated by uploading the key raw data onto the Research Data Deposit platform (www.researchdata.org.cn).

## Abbreviations

BLCA: Bladder urothelial carcinoma
IRGs: Inflammation-related genes
OS: Overall survival
TME: Tumor microenvironment
KEGG: Kyoto encyclopedia of genes and genomes
GSEA: Gene set enrichment analysis
AUC: Area under the curve
ANOVA: One-way analysis of variance
qPCR: Real-time quantitative polymerase chain reaction
GO: Gene ontology analysis
FDR: False discovery rate
HR: Hazard ratio
ssGSEA: Single sample GSEA
TAMs: Tumor-associated macrophages

## References

[1] Xia C, Dong X, Li H, Cao M, Sun D, He S (2022). Cancer statistics in China and United States, 2022: Profiles, trends, and determinants. Chin Med. J. (Engl.).

[2] Dobruch J, Oszczudlowski M (2021). Bladder cancer: Current challenges and future directions. Medicina (Kaunas).

[3] Hanahan D (2022). Hallmarks of cancer: New dimensions. Cancer Discov..

[4] Michaud DS (2007). Chronic inflammation and bladder cancer. Urol. Oncol..

[5] Cumberbatch MGK, Jubber I, Black PC, Esperto F, Figueroa JD, Kamat AM (2018). Epidemiology of bladder cancer: A systematic review and contemporary update of risk factors in 2018. Eur. Urol..

[6] Ohshima H, Tatemichi M, Sawa T (2003). Chemical basis of inflammation-induced carcinogenesis. Arch. Biochem. Biophys..

[7] Hanahan D, Coussens LM (2012). Accessories to the crime: Functions of cells recruited to the tumor microenvironment. Cancer Cell.

[8] Greten FR, Grivennikov SI (2019). Inflammation and cancer: Triggers, mechanisms, and consequences. Immunity.

[9] Singh R, Mishra MK, Aggarwal H (2017). Inflammation, immunity, and cancer. Mediat. Inflamm..

[10] Zheng J, Peng L, Zhang S, Liao H, Hao J, Wu S (2023). Preoperative systemic immune-inflammation index as a prognostic indicator for patients with urothelial carcinoma. Front. Immunol..

[11] Liu Q, You B, Meng J, Huang C-P, Dong G, Wang R (2022). Targeting the androgen receptor to enhance NK cell killing efficacy in bladder cancer by modulating ADAR2/circ_0001005/PD-L1 signaling. Cancer Gene Ther..

[12] Colaprico A, Silva TC, Olsen C, Garofano L, Cava C, Garolini D (2016). TCGAbiolinks: An R/bioconductor package for integrative analysis of TCGA data. Nucleic Acids Res..

[13] Sjödahl G, Lauss M, Lövgren K, Chebil G, Gudjonsson S, Veerla S (2012). A molecular taxonomy for urothelial carcinoma. Clin. Cancer Res..

[14] Mariathasan S, Turley SJ, Nickles D, Castiglioni A, Yuen K, Wang Y (2018). TGFβ attenuates tumor response to PD-L1 blockade by contributing to exclusion of T cells. Nature.

[15] Bhattacharya A, Hamilton AM, Furberg H, Pietzak E, Purdue MP, Troester MA (2021). An approach for normalization and quality control for NanoString RNA expression data. Brief Bioinform..

[16] Meng J, Lu X, Zhou Y, Zhang M, Ge Q, Zhou J (2021). Tumor immune microenvironment-based classifications of bladder cancer for enhancing the response rate of immunotherapy. Mol. Ther. Oncol..

[17] Arthur JC, Perez-Chanona E, Mühlbauer M, Tomkovich S, Uronis JM, Fan TJ (2012). Intestinal inflammation targets cancer-inducing activity of the microbiota. Science.

[18] Panigrahy D, Gartung A, Yang J, Yang H, Gilligan MM, Sulciner ML (2019). Preoperative stimulation of resolution and inflammation blockade eradicates micrometastases. J. Clin. Investig..

[19] Rothwell PM, Wilson M, Price JF, Belch JF, Meade TW, Mehta Z (2012). Effect of daily aspirin on risk of cancer metastasis: A study of incident cancers during randomised controlled trials. Lancet.

[20] Yu JI, Park HC, Yoo GS, Paik SW, Choi MS, Kim HS (2020). Clinical significance of systemic inflammation markers in newly diagnosed, previously untreated hepatocellular carcinoma. Cancers (Basel).

[21] Qiu C, Shi W, Wu H, Zou S, Li J, Wang D (2021). Identification of molecular subtypes and a prognostic signature based on inflammation-related genes in colon adenocarcinoma. Front. Immunol..

[22] Zhang S, Li X, Tang C, Kuang W (2021). Inflammation-related long non-coding RNA signature predicts the prognosis of gastric carcinoma. Front. Genet..

[23] Elinav E, Nowarski R, Thaiss CA, Hu B, Jin C, Flavell RA (2013). Inflammation-induced cancer: Crosstalk between tumours, immune cells and microorganisms. Nat. Rev. Cancer.

[24] Diakos CI, Charles KA, McMillan DC, Clarke SJ (2014). Cancer-related inflammation and treatment effectiveness. Lancet Oncol..

[25] Nabavizadeh R, Bobrek K, Master VA (2020). Risk stratification for bladder cancer: Biomarkers of inflammation and immune activation. Urol. Oncol..

[26] Gakis G (2014). The role of inflammation in bladder cancer. Adv. Exp. Med. Biol..

[27] Liang T, Wu X, Wang L, Ni Z, Fan Y, Wu P (2023). Clinical significance and diagnostic value of QPCT, SCEL and TNFRSF12A in papillary thyroid cancer. Pathol. Res. Pract..

[28] Lian M, Cao H, Baranova A, Kural KC, Hou L, He S (2020). Aging-associated genes TNFRSF12A and CHI3L1 contribute to thyroid cancer: An evidence for the involvement of hypoxia as a driver. Oncol. Lett..

[29] Wu J, Wan F, Sheng H, Shi G, Shen Y, Lin G (2017). NR1H3 expression is a prognostic factor of overall survival for patients with muscle-invasive bladder cancer. J. Cancer.

[30] Sun Y, Jin J, Jing H, Lu Y, Zhu Q, Shu C (2021). ITIH4 is a novel serum biomarker for early gastric cancer diagnosis. Clin. Chim. Acta.

[31] Zhang M, Zeng L, Peng Y, Fan B, Chen P, Liu J (2021). Immune-related genes LAMA2 and IL1R1 correlate with tumor sites and predict poor survival in pancreatic adenocarcinoma. Future Oncol..

[32] Takeuchi H, Yokota A, Ohoka Y, Iwata M (2011). Cyp26b1 regulates retinoic acid-dependent signals in T cells and its expression is inhibited by transforming growth factor-β. PLoS ONE.

[33] Kamoun A, de Reyniès A, Allory Y, Sjödahl G, Robertson AG, Seiler R (2020). A consensus molecular classification of muscle-invasive bladder cancer. Eur. Urol..

[34] Lu X, Meng J, Su L, Jiang L, Wang H, Zhu J (2021). Multi-omics consensus ensemble refines the classification of muscle-invasive bladder cancer with stratified prognosis, tumour microenvironment and distinct sensitivity to frontline therapies. Clin. Transl. Med..

[35] Wang J, Shen C, Dong D, Zhong X, Wang Y, Yang X (2021). Identification and verification of an immune-related lncRNA signature for predicting the prognosis of patients with bladder cancer. Int. Immunopharmacol..

[36] Wu Y, Zhang L, He S, Guan B, He A, Yang K (2020). Identification of immune-related LncRNA for predicting prognosis and immunotherapeutic response in bladder cancer. Aging (Albany NY).

[37] Jiang W, Zhu D, Wang C, Zhu Y (2020). An immune relevant signature for predicting prognoses and immunotherapeutic responses in patients with muscle-invasive bladder cancer (MIBC). Cancer Med..

[38] Yunna C, Mengru H, Lei W, Weidong C (2020). Macrophage M1/M2 polarization. Eur. J. Pharmacol..

[39] Farhood B, Najafi M, Mortezaee K (2019). CD8(+) cytotoxic T lymphocytes in cancer immunotherapy: A review. J. Cell. Physiol..

[40] Pfister C, Gravis G, Fléchon A, Chevreau C, Mahammedi H, Laguerre B (2022). Dose-dense methotrexate, vinblastine, doxorubicin, and cisplatin or gemcitabine and cisplatin as perioperative chemotherapy for patients with nonmetastatic muscle-invasive bladder cancer: Results of the GETUG-AFU V05 VESPER trial. J. Clin. Oncol..

[41] Pfister C, Gravis G, Fléchon A, Soulié M, Guy L, Laguerre B (2021). Randomized phase III trial of dose-dense methotrexate, vinblastine, doxorubicin, and cisplatin, or gemcitabine and cisplatin as perioperative chemotherapy for patients with muscle-invasive bladder cancer. Analysis of the GETUG/AFU V05 VESPER trial secondary endpoints: Chemotherapy toxicity and pathological responses. Eur. Urol..

[42] Vaughn DJ (2000). Paclitaxel and carboplatin in bladder cancer: Recent developments. Eur. J. Cancer.

[43] Jiménez-Guerrero R, Gasca J, Flores ML, Pérez-Valderrama B, Tejera-Parrado C, Medina R (2018). Obatoclax and paclitaxel synergistically induce apoptosis and overcome paclitaxel resistance in urothelial cancer cells. Cancers (Basel).

[44] Bellmunt J, von der Maase H, Mead GM, Skoneczna I, De Santis M, Daugaard G (2012). Randomized phase III study comparing paclitaxel/cisplatin/gemcitabine and gemcitabine/cisplatin in patients with locally advanced or metastatic urothelial cancer without prior systemic therapy: EORTC intergroup study 30987. J. Clin. Oncol..

[45] Mitin T, Hunt D, Shipley WU, Kaufman DS, Uzzo R, Wu CL (2013). Transurethral surgery and twice-daily radiation plus paclitaxel-cisplatin or fluorouracil-cisplatin with selective bladder preservation and adjuvant chemotherapy for patients with muscle invasive bladder cancer (RTOG 0233): A randomised multicentre phase 2 trial. Lancet Oncol..

