# Supplementary material for: Identification of an inflammation-related risk signature for prognosis and immunotherapeutic response prediction in bladder cancer
Source: Sci Rep. 2024 Jan 12;14:1216. doi: 10.1038/s41598-024-51158-9 (PMC10786915; doi:10.1038/s41598-024-51158-9)
Supplement: Supplementary file 1 — Supplementary Tables. [file 41598_2024_51158_MOESM1_ESM.pdf]

Identification of an inflammation-related risk signature for prognosis and immunotherapeutic response prediction in bladder cancer

Supplementary Tables

Table S1. Clinical information for the TCGA-BLCA cohort.

		TCGA-BLCA cohort	
Covariates		Primary tumors (n=411)	Normal tissues (n=19)
Age		68.06±10.58	69.89±11.31
Gender			
	female	108 (26.28%)	9 (47.37%)
	male	303 (73.72%)	10 (52.63%)
pT stage			
	pT1	4 (0.97%)	0
	pT2	119 (28.95%)	5 (26.32%)
	pT3	195 (47.45%)	11 (57.89%)
	pT4	59 (14.36%)	3 (15.79%)
	pTx	34 (8.27%)	0
pN stage			
	pN0	238 (57.91%)	11 (57.89%)
	pN1	46 (11.19%)	3 (15.79%)
	pN2	76 (18.49%)	5 (26.32%)
	pN3	8 (1.95%)	0
	pNx	43 (10.46%)	0
Metastasis			
	M0	199 (48.42%)	10 (52.63%)
	M1	11 (2.68%)	0
	Mx	201 (48.91%)	9 (47.37%)
Pathological grade			
	Low	21 (5.11%)	0
	High	387 (94.16%)	19 (100.0%)
	Unknow	3 (0.73%)	0
Clinical stage			
	Stage I	3 (0.73%)	0
	Stage II	130 (31.63%)	4 (21.05%)
	Stage III	141 (34.31%)	7 (36.84%)
	Stage IV	135 (32.85%)	8 (42.11%)

Table S2. The list of 2343 inflammation-related genes.

**Inflammation-related genes**									
ABCA1	CALCRL	CMKLR1	FPR1	IFNGR2	IRF7	MEP1A	P2RX7	RHOG	SLC7A1
ABI1	CCL17	CSF1	FZD5	IL10	ITGA5	MET	P2RY2	RIPK2	SLC7A2
ACVR1B	CCL2	CSF3	GABBR1	IL10RA	ITGB3	MMP14	PCDH7	RNF144B	SPHK1
ACVR2A	CCL20	CSF3R	GCH1	IL12B	ITGB8	MSR1	PDE4B	ROS1	SRI
ADM	CCL22	CX3CL1	GNA15	IL15	KCNA3	MXD1	PDPN	RTP4	STAB1
ADORA2B	CCL24	CXCL10	GNAI3	IL15RA	KCNJ2	MYC	PIK3R5	SCARF1	TACR1
ADRM1	CCL5	CXCL11	GP1BA	IL18	KCNMB2	NAMPT	PLAUR	SCN1B	TACR3
AHR	CCL7	CXCL6	GPC3	IL18R1	KIF1B	NDP	PROK2	SELE	TAPBP
APLNR	CCR7	CXCL9	GPR132	IL18RAP	KLF6	NFKB1	PSEN1	SELL	TIMP1
AQP9	CCRL2	CXCR6	GPR183	IL1A	LAMP3	NFKBIA	PTAFR	SELENOS	TLR1
ATP2A2	CD14	CYBB	HAS2	IL1B	LCK	NLRP3	PTGER2	SEMA4D	TLR2
ATP2B1	CD40	DCBLD2	HBEGF	IL1R1	LCP2	NMI	PTGER4	SERPINE1	TLR3
ATP2C1	CD48	EBI3	HIF1A	IL2RB	LDLR	NMUR1	PTGIR	SGMS2	TNFAIP6
AXL	CD55	EDN1	HPN	IL4R	LIF	NOD2	PTPRE	SLAMF1	TNFRSF1B
BDKRB1	CD69	EIF2AK2	HRH1	IL6	LPAR1	NPFFR2	PVR	SLC11A2	TNFRSF9
BEST1	CD70	EMP3	ICAM1	IL7R	LTA	OLR1	RAF1	SLC1A2	TNFSF10
BST2	CD82	ADGRE1	ICAM4	CXCL8	LY6E	OPRK1	RASGRP1	SLC28A2	TNFSF15
BTG2	CDKN1A	EREG	ICOSLG	INHBA	LYN	OSM	RELA	SLC31A1	TNFSF9
C3AR1	CHST2	F3	IFITM1	IRAK2	MARCO	OSMR	RGS1	SLC31A2	TPBG
C5AR1	CLEC5A	FFAR2	IFNAR1	IRF1	MEFV	P2RX4	RGS16	SLC4A4	VIP
TP53	ERBB2	SOD1	AIF1	CXCL2	HMGB1	PYCARD	IRGM	HAVCR2	CX3CR1
STAT3	EGR1	GJA1	VEGFA	MAPT	S100A8	ABCC2	CDH1	EP300	SFRP1
EGFR	TGFB1	CD44	LEP	PRKAA1	CXCL12	SMAD4	ANGPT2	VDR	PPARA
MYD88	SOD2	NR4A2	GATA3	GDF15	GSTP1	CD4	SLC11A1	MPO	TLR9
PPARGC1A	ADIPOQ	TREM2	CASP3	CCND1	CD36	MEF2C	SYK	IFNB1	IL37
CREB1	IL4	HSPA5	ZFP36L1	MMP9	BAD	PPARG	CXCL3	INS	CRP
TLR4	SIRT1	CFTR	G6PD	CYP1A1	CCL18	CCL26	FOXO1	MAPK3	DDIT4
PTGS2	IL23R	IL2	EPO	CYP2D6	JUN	EGR2	CCL11	AQP1	OGG1
AKT1	APOE	LCN2	HAMP	CCL19	LRRK2	PTK2B	TNFSF4	VNN1	TCIRG1
HLA-DRB1	CCL3	HMOX1	FYN	IL13	CYBA	ESR1	F7	CCL1	FOSL1
TNF	COL1A1	SNCA	APOB	CDKN1B	IRF5	ANXA1	TGFBR2	MDK	ASS1
MAPK14	MAPK1	STING1	CCR1	ARG1	CBL	PON1	CAV1	CASR	TNFRSF1A
ATF4	NFE2L2	HLA-B	HLA-DQA1	ALOX5	CCR2	DNMT3B	STAT5B	RHOA	GCLC
PTEN	MTOR	FCGR2B	IFNL3	VCAM1	CHUK	PARK7	PIK3CA	FOXP3	EZH2
IL2RA	SRC	IFNG	NR4A1	SLC6A4	GPX1	IGF1	WNT5A	XBP1	HLA-DQB1
STAT1	NOS2	CCL4	HSPD1	IL17A	IL27	CREM	BRCA2	AR	IL12A
BCL2	FOS	SMAD3	RETN	CCR5	TPMT	ABCB1	IL1RL1	BAX	ZC3H12A
MDM2	JAK2	THBS1	NQO1	PRKCD	IL21	CLDN1	CAMP	CASP8	HLA-A
KRAS	BRAF	CEBPB	TNFAIP3	NOTCH1	CTNNB1	HLA-DRA	PARP1	WWOX	TRPV1
APP	STAR	HSF1	CAT	CXCR4	S100A9	FOXO3	MTHFR	AGER	CRH
PRKN	ATR	FGF23	CREB5	SDC1	FOXP1	TNFSF11	TGFB2	MAP2K1	PTK2

HDAC4	CCL15	BCL2L1	SLC9A1	NOX4	BCL3	SULT1A1	SAMHD1	HSPA4	MITF
CXCR2	IL1RN	MUC1	SOCS1	SIRPA	APEX1	STAT4	COL2A1	BIRC2	ADAM8
MYOC	SPARC	BCL2A1	NR1H4	PTX3	ADRB2	NOS1	SMARCA4	WT1	IDO1
APOA1	NUPR1	HLA-G	KLF2	S100A12	HLA-DPB1	CALR	IRS1	USF1	H2AX
ELANE	PTN	CXCL13	F2RL1	PTPN2	ACE2	FADS2	CRYAB	NR1D1	CGAS
GRN	F2	LMNA	TGM2	NR1H3	EDNRB	HNRNPD	MCL1	TIMP2	ABCD1
SLC2A1	IGF1R	GSK3B	B2M	IKBKB	CYP3A5	CDKN2A	APOA2	NR3C2	CXCL16
ADAM17	COMT	BAP1	SOX9	CFLAR	TIRAP	AGTR2	CPT1A	ANG	DNASE1
NFAT5	XIAP	PDCD1	RYR1	SESN2	DEFB4A	CD6	CD24	ABCG2	BIRC5
GHRL	ALK	HDAC1	MCPH1	HNF4A	PRKCB	GIP	FLT1	NLRP7	PAK1
MAPK8	RET	NTRK1	CNR2	NOD1	IFNA2	KDM1A	DPP4	PPP5C	S100A7
KLF4	SREBF1	PRKCQ	TAC1	IRF3	TICAM1	S100B	FN1	MST1R	GSDMD
TRPV4	CD40LG	CXCL1	TGFBR1	IGFBP2	HDAC6	ACOD1	PRKAA2	MAFB	BCHE
OXT	CALCA	TWIST1	LYZ	RPS19	TNNT2	GSTT1	TRIB1	SLPI	SFTPD
LGALS9	AGT	MECP2	TLR8	DEFB1	TNFRSF6B	KDR	SERPINA1	SQSTM1	PROC
RUNX1	TNFRSF12A	BTK	CXCL5	CCR3	IL24	MAVS	POLB	CCL13	RPS3
TNC	ADORA2A	POSTN	ALOX15	IL10RB	CD38	IL17RA	WRN	GGT1	NEDD4L
BCL2L11	NRAS	PINK1	ALPL	BCL6	PLA2G2A	FGFR3	EPAS1	IGF2R	CD163
FASLG	SMAD7	HP	ATG16L1	RPS6KB1	TXNIP	F11R	CTSB	CIITA	ACTA1
IFIH1	LPA	SCGB1A1	UCP2	DHX9	TET2	LOX	CXCR1	HTR2A	IL11
DDX58	DUSP1	PIK3CG	FAS	MICA	MSTN	BCL10	GPX4	RELB	TXN
GBA	LPL	ETS1	HFE	PRDX2	NLRP1	HYAL1	SAA1	CD209	ACTC1
SPP1	ITGA2	CLU	TRAF3IP2	FADS1	ITGB2	MSH6	VIM	CFH	IL32
MMP2	HCK	IER3	CYP19A1	TREM1	LGALS3	CHGA	BDKRB2	RNASE3	NCF1
CCN2	GCLM	SIRT2	FGF21	SELP	AIM2	TNFAIP2	IFNAR2	DEFA5	SCNN1B
ADA	CASP9	MICB	IL17F	MAP3K5	MAPK7	APOA5	TNFSF13B	CD47	RBPJ
IKBKG	NUDT15	RORA	LGALS1	XRCC1	NPPB	PTPN1	DPYSL5	SOX5	NRP1
NOS3	GNAS	IL6ST	F2R	TNIP1	LILRB1	ATF3	MX1	ZBP1	ELAVL1
CARD9	MBL2	PKD2	TRAF6	ESR2	IRAK1	TNFRSF11A	PGF	PLP1	ATG7
PTPN11	RAC1	OXTR	RIPK1	BMPR2	SLC30A8	REG1A	NPPA	TBXA2R	CD244
CHI3L1	CHRNA7	STK11	IFI16	ITGB1	MMP1	LY96	FABP4	ARRB1	THBD
PTPN22	GHSR	TSPO	SMAD2	PLAU	CRHR1	XCL1	JAK3	CD74	CTSS
HSP90AA1	ZFP36	CCR6	C3	SMPD1	CRHBP	EPHA2	TSLP	AVP	TFPI
YAP1	CASP1	PDGFRB	PCSK9	IL6R	SOCS3	BACE1	GAS6	APBB1IP	GAL
ACE	PPARD	CRTC3	F8	E2F1	ADCYAP1	HYAL2	TGFBI	CD28	IGFBP3
RB1	TLR7	APOBEC3G	IL33	REL	TBK1	FLG	FCGR3B	TNNI3	ITGA4
CYP2C8	FCGR3A	ADAMTS13	BDNF	IGF2	C5	ZMIZ1	GPI	PTPRC	PRKCA
PF4	MIF	DISC1	HSPA1A	PRKD1	FGFR1	TEK	YY1	ATG5	SPRED1
HLA-C	NR3C1	BMP2	CCL4L1	TRPA1	HSPB1	FADD	FGB	ADIPOR2	AQP3
CCL23	ENG	APC	FCGR2A	PRKCZ	ABCC1	FGF2	PDCD4	BTNL2	PGR
OPRM1	MAPKAPK2	TP63	KIT	ERAP1	TLR5	ADAR	ITGAL	CASP4	KDM5B
DAB2IP	BMP7	CLEC7A	IFNA1	CCL21	IRF8	CDKAL1	CARD8	AREG	IL1R2
IL23A	AGTR1	TREX1	TNFRSF14	NOTCH2	DRD3	AKT2	IDH1	BRD4	RORC
CD274	CREBBP	PDGFRA	RGCC	MMP3	SP1	TNFRSF4	LRP1	JAG1	RCAN1

**IL22**	**LBP**	**FHIT**	**HGF**	**GSTM1**	**STAT5A**	**STAT6**	**HMGB2**	**PTGS1**	**KEAP1**
**CSF2**	**RARRES2**	**CTLA4**	**IL5**	**KDM6B**	**BSG**	**NR2F2**	**CD81**	**IKZF1**	**NOX1**
**SHH**	**SERPINF1**	**LTF**	**FPR2**	**ITLN1**	**VWF**	**HSP90AB1**	**ROCK2**	**TIMP3**	**GATA2**
**CNR1**	**PTGES**	**TNFRSF11B**	**CYLD**	**DEFA1**	**CEBPA**	**MAP2K4**	**PLCG2**	**CSF1R**	**PDGFB**
**GPER1**	**UCN**	**PRKDC**	**TLR6**	**BMP6**	**PKD1**	**CXCR3**	**LITAF**	**GPR65**	**POMC**
**ITGAV**	**SNAI1**	**ALOX5AP**	**FGF10**	**TOR3A**	**ENPP3**	**DLL4**	**DLL1**	**SERPINA3**	**PI3**
**PIK3CD**	**TGFB3**	**IL1F10**	**ANO1**	**SFRP2**	**HSD11B1**	**HSPA1B**	**F12**	**UCN3**	**SLC6A8**
**PNPLA3**	**INPP5D**	**BMP4**	**STC1**	**SREBF2**	**HDAC3**	**AOC1**	**LSP1**	**CMA1**	**CCNE1**
**CLOCK**	**MYLK**	**LRP5**	**IFIT1**	**MAP3K1**	**ITGB6**	**FUT4**	**FFAR4**	**IGFBP5**	**HDAC7**
**SUMO1**	**MT2A**	**APOD**	**TRAF3**	**ACP5**	**MED1**	**SCARB1**	**ROR2**	**HPGD**	**MID1**
**NR5A2**	**EGF**	**CD86**	**EPHX1**	**MALT1**	**CDO1**	**RUNX3**	**RAPGEF3**	**ANGPTL4**	**GALC**
**ISG15**	**TPM1**	**IL9**	**STAB2**	**CDC37**	**FOXA1**	**SPI1**	**CNTF**	**C4A**	**ADAMTS12**
**IL36G**	**DAPK1**	**HSP90B1**	**TAB2**	**IRAK3**	**TAGAP**	**NRG1**	**GNA12**	**HK1**	**TRIM32**
**PLA2G7**	**HSPA8**	**YBX1**	**ALDH2**	**ABCG1**	**IL12RB1**	**CEACAM1**	**ORM1**	**GART**	**WNT7A**
**SLC22A5**	**IL19**	**YWHAQ**	**ERAP2**	**HDAC9**	**PRDX5**	**TFF1**	**ARRB2**	**SPN**	**C1QA**
**LILRB4**	**PDE4D**	**CYP11B2**	**SNAI2**	**GHRHR**	**KLF5**	**APOBEC3A**	**VTCN1**	**HDAC5**	**F10**
**MAP2K6**	**IL1RL2**	**THADA**	**CTSL**	**PHB**	**CLN3**	**NOTCH3**	**TTR**	**ESM1**	**APOL1**
**GAPDH**	**ADRB1**	**FCGR1A**	**HNMT**	**NLRP2**	**CD5**	**PRDX1**	**CCL16**	**KAT7**	**COL18A1**
**IKBKE**	**CCR4**	**OPTN**	**NAT2**	**PDCD1LG2**	**KLK3**	**TIMP4**	**CYP24A1**	**PGLYRP1**	**FURIN**
**SPHK2**	**ZAP70**	**TCF4**	**YES1**	**CCL3L1**	**APLN**	**DCN**	**GZMB**	**CDK6**	**DIABLO**
**L1CAM**	**P2RX3**	**FTO**	**CD1D**	**CD46**	**PTGFR**	**ENO1**	**IL18BP**	**TYROBP**	**IL31RA**
**FOXF1**	**CDK5**	**CDC42**	**FEN1**	**APCS**	**IL12RB2**	**C1QBP**	**KIR3DL1**	**CCN1**	**ITGA3**
**KCNMA1**	**FOXM1**	**ANGPT1**	**SEMA3A**	**IL5RA**	**KDM6A**	**NLRC4**	**USP7**	**MFHAS1**	**BANK1**
**TNFRSF10B**	**HTT**	**MC1R**	**RBP4**	**TNFSF14**	**PDE2A**	**AGR2**	**SPRY4**	**S100A4**	**BABAM2**
**ROCK1**	**DEFB103B**	**PRF1**	**SATB2**	**NFKB2**	**ACKR1**	**TNFAIP8L2**	**METTL3**	**KARS1**	**LNPEP**
**FOSB**	**SERPINC1**	**SLIT2**	**SOST**	**GLP1R**	**KIAA1109**	**ROBO1**	**ERBB4**	**PRKCI**	**AHCY**
**MAPK13**	**TLR10**	**PIN1**	**TRIM27**	**DROSHA**	**ADCYAP1R1**	**SEMA7A**	**CD160**	**KRT8**	**TOR1A**
**DNM1L**	**MKI67**	**PDPK1**	**INAVA**	**PPP2CA**	**APOA4**	**FANCD2**	**HRH4**	**ERG**	**MAS1**
**CYP27B1**	**CHRNA4**	**DMBT1**	**IL7**	**CDKN2B**	**MC4R**	**C4B**	**SPON1**	**ARSB**	**GNAO1**
**AHSG**	**PPBP**	**TRPC6**	**EGLN1**	**PROM1**	**NCL**	**MMP13**	**TOP1**	**SLC7A5**	**GSDMB**
**ELN**	**IL16**	**NR4A3**	**EIF4G1**	**TNFSF12**	**NOTCH4**	**NR1H2**	**IFNA21**	**APOC3**	**FFAR3**
**CALM3**	**CTSG**	**HBB**	**SMAD1**	**PTGDR2**	**NPAS3**	**PECAM1**	**STIM1**	**PRTN3**	**GSK3A**
**ITCH**	**MAP3K8**	**ADORA1**	**GREM1**	**TF**	**DKK1**	**PAPPA**	**ENPP1**	**EFNB1**	**TEC**
**NKX3-1**	**CHRNB2**	**AZU1**	**APOC1**	**NGF**	**KAT2B**	**CARD14**	**MUC16**	**IL34**	**POU2F1**
**GCG**	**IRF6**	**FGG**	**MME**	**IL3**	**JAK1**	**PTGIS**	**CERS6**	**ADORA3**	**TGFA**
**UBE2L3**	**IL36RN**	**NR1I2**	**CCL8**	**LMNB1**	**CUX1**	**CD247**	**PRDM1**	**IKZF3**	**SCNN1A**
**PROCR**	**LACC1**	**FGR**	**FNDC5**	**MAP2K2**	**KRT18**	**AIRE**	**TNFSF13**	**NR0B2**	**HVCN1**
**HMGCR**	**SGMS1**	**SIRT6**	**CPEB4**	**NLRP6**	**SMPD3**	**NCOR2**	**NFATC2**	**NFIL3**	**REV3L**
**ADAM10**	**GLA**	**SIX1**	**CXCR5**	**PF4V1**	**TNFRSF10A**	**TBX5**	**CTSV**	**TRADD**	**PADI4**
**DBH**	**TYK2**	**IRS2**	**PKM**	**HNRNPA1**	**ACVR1**	**CITED2**	**GUCY2C**	**MTDH**	**SLAMF7**
**MAML2**	**CPB2**	**F11**	**MMP8**	**DLK1**	**ORMDL3**	**FASN**	**PRKRA**	**OCLN**	**SELPLG**
**IL20**	**NLRP10**	**IL25**	**CD34**	**NPY**	**HNF1A**	**SOD3**	**CCL25**	**ARMS2**	**LGMN**
**P2RY12**	**ALB**	**DUOX2**	**GLI1**	**CD80**	**FLNB**	**NGFR**	**SMAD6**	**MST1**	**NFKBIZ**
**CST3**	**TBX21**	**KL**	**AOC3**	**CSNK2A1**	**SSTR2**	**CD68**	**IRAK4**	**PER1**	**CBS**

TRAF2	AQP4	RXRA	ADCY7	DYSF	PLAT	LAT	BTRC	ADRB3	RNASE2
DEFA3	PLCG1	TSHR	HPSE	STK39	FXN	ADAM15	MAP3K14	ACTN3	IAPP
GBP1	F5	IFNL1	NCAM1	ITGAM	CDH5	CD19	SLC9A3	BMPR1B	FCN2
CD226	JAZF1	BIRC3	TFRC	WNT1	PLA2G4A	ZEB1	ITIH4	PIM1	ABCB4
DNMT1	TNFSF18	INHBB	CADM1	PPIA	PDYN	MAP3K7	NCR3	ICOS	KRT1
PTHLH	CD200	HSPA6	ORAI1	SETX	BACH2	RSAD2	CARD11	ARPC1B	APOH
NELL1	PTPN6	MTMR3	LILRB2	STAT2	PON2	CD84	ENPP2	CHIT1	RNASET2
PRL	IL36A	SCN9A	NT5E	ASIC1	SBNO2	RIPK3	CTSK	NONO	COMP
KLRK1	DEFA1B	MRC1	AFP	STS	NEDD9	CEACAM5	TNFRSF8	MAPK9	TRIM28
LEPR	NTRK3	NDRG1	ST6GAL1	KIR2DL1	ACKR3	IGFBP1	NLRP12	ZMPSTE24	AIMP1
PPM1D	BPIFA1	TRIM21	SOX4	WAS	ARG2	MX2	STMN1	RGS2	TNFRSF18
CYSLTR1	ERCC8	SRD5A2	SRR	ACAN	GFER	MAG	IL17D	ARID3A	ZNF268
CFL1	FLT4	IGF2BP3	RANBP2	CD5L	ATP6V0A2	CHIA	MACROD1	BVES	SLC38A9
TOLLIP	LAG3	PTBP1	FAM135B	DCLRE1B	FCRLA	CSRP3	LRBA	DUOXA1	TNK1
KLKB1	PLIN2	TRPM7	MCC	CASP6	XCR1	ABHD12	RPL13A	DDX3Y	DAGLB
UCN2	PPIF	NPTX2	NCSTN	EPRS1	KPNA6	AFAP1L2	NCS1	MAPK6	SCYL3
IL36B	THBS4	NUSAP1	PFKFB3	FSTL1	PDE4A	TIGIT	HCAR2	NMU	CDNF
LTB4R	CR2	CCN3	ADARB1	GGT5	PNOC	COMMD1	TRAF5	AKIRIN2	TMEM100
GC	RGS14	ALOX12	CEBPG	GPR55	SHARPIN	MGST2	IL22RA2	CHST1	BCL2L14
SMN1	RPS6KA2	GJB1	KHDRBS1	KLF9	HPX	CAMK2D	ITGAE	SETD6	NFAM1
TP53INP1	GNAI1	S100A10	CLDN3	CLDN8	LTC4S	PHLPP1	HCRTR1	TREML1	SELENOH
MYH9	SERPING1	LTBR	TFF3	FLI1	SULF1	FLT3LG	DEFB114	PARP14	SLC17A9
NPPC	SCUBE1	MAP2K3	CCL14	ASAH1	KRT17	PLA2G5	FCRL4	DPP9	PYDC1
FGF1	FGF7	PLK2	SLC2A9	CLDN5	PTPRK	SLIT3	LILRB3	SMC4	BAHD1
TNPO3	KRT16	IFI35	GSDMA	CD99	HNRNPA0	LTB	PLD4	TAOK1	NOSTRIN
C9orf72	MATN3	ASAH2	IPMK	HABP2	HLA-F	SAA2	GALNT17	USP25	CLEC2B
PIK3CB	UMOD	CR1	RGMA	CXADR	SLC26A3	ATRN	DNTT	LPIN2	ZNF598
IRF4	CGA	NRP2	CREB3L3	C1QTNF3	SLAMF8	PLA2G4C	PAG1	MTUS1	ATP11A
ASH1L	NFATC1	KLRG1	HNRNPA2B1	P2RX1	SULT2A1	ADM2	NT5C3A	MAFG	SLCO3A1
HMGA1	NTN1	ABCC6	IGFBP4	MMP26	TNFRSF17	CCDC88B	LIAS	GLP2R	LRRFIP2
NDFIP1	ETV6	GPR68	TBXAS1	VIPR1	BRD2	ATP8B1	CD93	LXN	VSTM1
ITPA	ACSL1	MYOCD	BST1	CYP2J2	POU1F1	CLEC4A	SERPINB1	SETD4	ANKZF1
PANX1	SHBG	PFN1	PSMA6	NPY5R	FGL2	AKAP5	CCS	TRIM23	ETV3
TIAM1	COPS5	PFKFB4	LILRA2	SP140	SDC2	ITGB7	NAPEPLD	TAX1BP1	ZDHHC2
SERPINH1	HAVCR1	LPIN1	EPX	PTGER1	NFATC3	TRAF4	CELA1	DCP1A	SLC39A10
FAAH	TRPC1	GRK2	PGLYRP2	MOG	PPP1R13L	TIFA	SIGLEC9	AGTRAP	HSH2D
SERPINB5	KITLG	CTSC	PADI2	ANPEP	BMX	NPR1	CHID1	C1QTNF1	TMIGD3
MGLL	PRMT5	SDC4	SCNN1G	CD96	SERPINA6	F2RL2	ALPI	DEFB104B	ART1
CEMIP	TYRO3	ITGAX	KCNJ5	IRF2	CYP4V2	MGAT5	GKN1	AKNA	APOO
P2RY6	LGALS3BP	NCOA4	CNP	PON3	PGRMC1	PGLYRP3	PLCB3	MTPN	MAPKBP1
CASP7	CXCL14	HMOX2	GIT1	OASL	UNC13D	VIPR2	ASIC2	NCR3LG1	MSMP
ITGA6	PTGER3	IL2RG	TET1	GPNMB	PLA2G10	IL17C	IL22RA1	DPP8	FIBCD1
KNG1	MLH3	DDX5	TXNRD1	PIAS3	SRGN	REG1B	PIK3R3	ACTR1A	RNF183
OTUB1	RPS6KA5	SLC5A1	LIPA	DOCK2	NTS	RHBDF2	NFX1	AOAH	TSPAN18

**SETD7**	**PNKD**	**TJP1**	**FREM1**	**NPSR1**	**DEFB104A**	**LILRA3**	**DIO3**	**TRIM55**	**LPCAT2**
**HRG**	**SGK1**	**CTSZ**	**MLKL**	**EFNB2**	**SLC2A5**	**TDO2**	**TRIM59**	**CBR3**	**NFKBID**
**MUC5AC**	**COMMD10**	**HCRT**	**PLD3**	**SARM1**	**SCG2**	**USP11**	**IL21R**	**FGF12**	**TREML4**
**PLG**	**RASSF5**	**JAM3**	**APPL2**	**CCL3L3**	**SIGIRR**	**LACRT**	**TFCP2**	**NLRP4**	**LRRC19**
**SRSF2**	**TRPM8**	**CAMK4**	**UBQLN4**	**PTGDS**	**PSMB4**	**PRSS8**	**FRK**	**TSPAN2**	**OCSTAMP**
**TMSB4X**	**SIGLEC5**	**HERC5**	**SERPINB2**	**FFAR1**	**MARCKS**	**VPS4A**	**TRIM8**	**EDIL3**	**GPR33**
**ECM1**	**DPEP1**	**UBR5**	**BPI**	**RBM4**	**TRIAP1**	**VDAC2**	**CHST4**	**SLC6A19**	**IGSF6**
**YWHAZ**	**AHCYL2**	**SOCS2**	**CDH3**	**PLD2**	**DAB2**	**NDST1**	**CD300A**	**ODAM**	**ZC3H12C**
**F13A1**	**CD59**	**TRAP1**	**CFP**	**BLNK**	**SHMT2**	**VPS4B**	**DEFB126**	**DUSP5**	**ARRDC4**
**NFATC4**	**GBP5**	**NR1D2**	**IP6K2**	**THPO**	**HAS1**	**CISH**	**TNIP3**	**PDZK1IP1**	**COX2**
**TNFSF8**	**RIT1**	**SATB1**	**FAP**	**CRB1**	**CEBPD**	**GPX2**	**TUSC2**	**BPIFB1**	**LRFN5**
**DUSP4**	**NFIA**	**ZP3**	**SERPINB3**	**REG4**	**TM9SF4**	**DHH**	**CUEDC2**	**TLE4**	**HARS1**
**HYAL3**	**RICTOR**	**PEBP1**	**HOMER1**	**KHSRP**	**LIME1**	**FSTL3**	**CISD2**	**PLA2G2E**	**C1QTNF12**
**ITGB4**	**SHMT1**	**ANKRD26**	**MASP2**	**ETS2**	**PTPN13**	**ICAM3**	**FLII**	**NEK7**	**HMGN2**
**DLD**	**CISD1**	**AMPD1**	**RAB8A**	**CD200R1**	**RNASE1**	**MMP25**	**RBCK1**	**TPST1**	**USP4**
**NLRX1**	**IFNL2**	**CIRBP**	**ADAM33**	**TERF2IP**	**SMPDL3B**	**ADA2**	**MGAT3**	**CCDC3**	**TMBIM6**
**FOXO4**	**EDNRA**	**PRKG1**	**ADAMTS7**	**ACP1**	**CDK19**	**TPSD1**	**TAB3**	**TMX1**	**TYMP**
**DDX21**	**PTGDR**	**HSD3B1**	**GPR4**	**GPS2**	**MAP3K9**	**DDT**	**NFASC**	**HDAC11**	**SOCS5**
**PBK**	**KCNN4**	**CCL27**	**SP3**	**ABCB11**	**NTF3**	**CXCL17**	**MAZ**	**SAA4**	**CD180**
**CD1A**	**UFL1**	**PRLR**	**ABCC3**	**ARHGEF4**	**SFRP5**	**INPP4B**	**NRROS**	**APOL3**	**COL7A1**
**ARF6**	**AFF2**	**VCAN**	**STAU1**	**SOX17**	**ADAMTS1**	**CAV2**	**RNF40**	**ICMT**	**DIO2**
**EEF1A1**	**ENO2**	**KLK6**	**CD63**	**TICAM2**	**NAALADL2**	**TRD**	**SPRY1**	**RFTN2**	**CTH**
**SF3B1**	**AMBP**	**CYP26B1**	**TXK**	**CDA**	**FEM1A**	**SRSF5**	**NLRP9**	**MYLK3**	**AZGP1**
**ACKR2**	**ANGPTL8**	**CHP1**	**MANF**	**DAP**	**ADGRB1**	**VSIR**	**HPR**	**PXK**	**CPEB1**
**ADGRE5**	**CYSLTR2**	**CSF2RB**	**USP47**	**U2AF1**	**ILF2**	**FMO1**	**ECRG4**	**SH2D2A**	**ECE1**
**CYP4F8**	**TANK**	**ADAMTS5**	**LGALS8**	**NCR1**	**TNFRSF25**	**GPR83**	**PPM1G**	**HAAO**	**PIM2**
**NLRC5**	**CP**	**CELF1**	**NTSR1**	**OTULIN**	**CHRFAM7A**	**CDK5R1**	**PYDC2**	**GLG1**	**ADGRE2**
**CTNND1**	**PRMT1**	**GLUL**	**SRSF3**	**CD151**	**CCL28**	**CHST8**	**ORM2**	**ITGAD**	**PELI1**
**ADCY3**	**ABCA3**	**MUC4**	**DDR1**	**PLA2G2D**	**S100A1**	**S1PR2**	**GPR31**	**RHBDD3**	**KRT7**
**IFNGR1**	**DUSP10**	**EIF2AK1**	**GSTO1**	**RO60**	**AP3B1**	**ADAMTS9**	**LGALS2**	**AMBN**	**FUT1**
**UBD**	**ISL1**	**PROS1**	**HSD3B2**	**CCKBR**	**TIAL1**	**MMP19**	**PYHIN1**	**BMP3**	**LDOC1**
**AICDA**	**APOM**	**NFE2L1**	**IL17B**	**DST**	**RNF2**	**LOXL3**	**MCOLN2**	**GPR15**	**SHPK**
**NFKBIL1**	**TSC22D3**	**ERBIN**	**HDGF**	**MMP10**	**LPCAT3**	**CAMK1D**	**MAP3K4**	**OSGIN1**	**CHGB**
**S1PR1**	**RHOC**	**NUCB2**	**PLA2G3**	**PARP9**	**ASIC3**	**TACR2**	**SCN11A**	**THOC5**	**BRAP**
**A2M**	**PYY**	**THY1**	**CFI**	**HCN2**	**TRPV3**	**FCN1**	**TAC4**	**PDLIM2**	**LPAR3**
**GCKR**	**IFIT3**	**NAT1**	**SFTPC**	**METRNL**	**MYO9B**	**FERMT3**	**BHLHE41**	**TRIL**	**PYDC5**
**MRTFA**	**ADIPOR1**	**FCAR**	**SLC23A2**	**HJV**	**ACLY**	**RSF1**	**VNN2**	**PLXNB2**	**PLA2G4B**
**ENTPD1**	**MUC2**	**SERPINB9**	**AMY1A**	**LPXN**	**SGF29**	**CAVIN1**	**GRK6**	**SNAPIN**	**GPR32**
**MS4A2**	**VTN**	**SLC40A1**	**NOL3**	**PDCD5**	**BCL11B**	**TRIB2**	**ABCD2**	**MUC17**	**LILRA5**
**FANCA**	**PSTPIP1**	**CASP5**	**PSMD10**	**LY75**	**SEC61A1**	**IL17RC**	**C2CD4B**	**OSCAR**	**IL17RE**
**GPC5**	**CCN4**	**TMPRSS2**	**GPBAR1**	**LYVE1**	**CLEC16A**	**SUPT5H**	**EN1**	**AFAP1**	**AREL1**
**CD83**	**ACHE**	**CCNT1**	**CTSW**	**OPRL1**	**DCSTAMP**	**BRD1**	**TMCO1**	**MAP3K12**	**PBX3**
**S1PR3**	**SELENOP**	**KLF14**	**CKM**	**CEACAM6**	**STK26**	**PI4KA**	**CNTN1**	**ZNF580**	**SPATA2**
**MSN**	**RARB**	**VPS35**	**SERPINA12**	**IL3RA**	**GPSM3**	**BCL6B**	**XYLT1**	**PANX2**	**THEMIS2**

CD27	FGF19	GAB2	UBE2N	CDK8	EGFL7	NUCKS1	H2BC1	ADAMDEC1	C1QTNF9
FUT7	ZFP90	TTC7A	NLRC3	COL6A5	CLDN2	SERPINB4	NEUROG3	GDE1	TRG
SIGMAR1	ZYX	CASP12	GRM3	OTUD7B	XCL2	LY86	LST1	PTGR1	MAGI2
IL1RAP	HOXA10	MVK	SUCNR1	RAB1A	DDX39B	GFRA1	FPR3	SPNS2	ZBTB20
PER2	MERTK	IL13RA2	DDAH1	P2RY11	LTA4H	MLANA	C2CD4A	SERPINB13	PJA2
NAIP	B4GALT1	PRG4	ZNF804A	NOX5	MMP11	TKT	ESRP1	OBP2A	MAP4K4
SLC26A4	NES	PTPA	CFB	EOMES	SCN10A	SERPIND1	NKRF	CH25H	MEP1B
HSPG2	MTTP	NINJ1	PARP4	SPINT2	SCYL1	HEY2	CNTN2	ZNF281	SLC23A1
SLC39A11	PEAR1	CTNNBIP1	SLC39A14	FBLN5	ABCF1	TIE1	FNDC4	PIK3AP1	PLAG1
RHOB	MACIR	TPSAB1	SPINK5	FXR1	SLC22A3	CLCA1	ANKRD2	CARD18	HSPE1
APPL1	NR2C2	IGF2BP1	AKR1C1	FBXO32	PSMA7	TRIM33	KYNU	NUP85	WFDC1
RPS6KA4	SPRED2	GZMA	FOLR2	HIF3A	LMAN1	PBXIP1	DUOXA2	CRLF1	KLK1
FCER2	KSR1	XDH	TLN1	TNIP2	CCR8	RNF31	ALPK1	ACER3	DENND1B
DUOX1	MCAM	PROX1	CLEC4E	PSMB8	PLAA	GDF11	ATG12	CD300C	SIGLEC1
PDGFA	REG3A	USP18	ACO1	VASH1	SIGLEC10	GPRC5B	SLC6A9	PLGRKT	DRGX
IL31	LIN28A	TRAF1	C5AR2	MASP1	CTHRC1	RLN1	IL20RB	PNMA1	DNASE1L3
CTSD	LUM	LAMTOR5	PIEZO1	SFTPA1	CTSH	LTB4R2	FST	PSEN2	RNF213
MAP2K5	DCD	ELF3							

Table S3. Fifty-nine upregulated and 426 downregulated differentially expressed inflammation-related genes were identified.

Gene	P-value	LogFC	threshold	Gene	P-value	LogFC	threshold
ACKR3	9.30E-17	1.248	Up	ABCA1	1.57E-49	-2.953	Down
ANGPT2	3.05E-47	2.169	Up	ABCB1	1.05E-20	-1.874	Down
BIRC5	1.13E-13	1.571	Up	ABCC3	4.52E-37	-2.903	Down
BSG	4.13E-18	1.109	Up	ABCG1	8.06E-20	-1.958	Down
CAV1	1.57E-13	1.008	Up	ACKR1	1.51E-20	-2.089	Down
CCNE1	1.66E-22	1.434	Up	ACO1	2.75E-17	-1.321	Down
CD36	3.81E-10	1.72	Up	ACTA1	3.03E-27	-1.629	Down
CD63	6.30E-20	1.211	Up	ADAM33	1.66E-37	-1.994	Down
CD82	1.67E-06	1.024	Up	ADAMTS1	2.55E-26	-2.016	Down
CD83	1.05E-05	1.299	Up	ADAMTS12	2.61E-09	-1.079	Down
CDKN2A	7.28E-15	1.455	Up	ADCY7	2.11E-31	-1.546	Down
CTSV	2.84E-28	1.261	Up	ADGRE2	5.60E-21	-1.066	Down
DLK1	0.00833	1.994	Up	ADORA3	1.04E-17	-1.646	Down
DLL4	6.30E-29	1.573	Up	AGTR1	9.01E-13	-1.488	Down
DUSP5	0.00471	1.167	Up	AIF1	2.30E-05	-1.039	Down
E2F1	1.55E-21	1.29	Up	ALB	2.01E-05	-1.061	Down
TNFRSF12A	6.63E-19	2.121	Up	ALOX5	5.20E-45	-2.652	Down
IL32	2.03E-06	1.265	Up	ALOX5AP	6.51E-21	-2.184	Down

NUCB2	6.09E-16	1.1	Up	ALPL	9.54E-10	-1.562	Down
FGFR1	3.58E-08	1.052	Up	ANGPTL4	1.09E-06	-1.147	Down
HCN2	1.22E-12	1.347	Up	ANKZF1	7.33E-23	-1.172	Down
MMP11	4.78E-15	1.142	Up	ANPEP	6.00E-27	-2.366	Down
NPTX2	6.94E-31	4.168	Up	AOC3	1.71E-08	-1.953	Down
VWF	4.73E-32	2.467	Up	APBB1IP	2.05E-22	-1.478	Down
MVK	1.31E-10	1.552	Up	APOBEC3G	1.45E-23	-1.702	Down
FOXM1	1.12E-20	1.733	Up	APOC1	1.52E-17	-2.922	Down
GAPDH	1.23E-20	1.569	Up	APOD	3.34E-18	-2.307	Down
SPARC	4.04E-14	1.596	Up	APOE	7.40E-09	-1.668	Down
HMGCR	5.29E-05	1.336	Up	APOL1	8.39E-05	-1.036	Down
THBS4	1.71E-21	1.457	Up	APOL3	3.26E-29	-1.641	Down
LMNB1	1.20E-15	1.036	Up	ARRB1	3.39E-15	-1.119	Down
PFKFB4	2.83E-13	1.202	Up	ARRB2	3.78E-40	-1.961	Down
SDC1	3.68E-12	1.556	Up	ASH1L	1.72E-25	-1.101	Down
SLC2A1	4.92E-10	1.152	Up	ATP11A	8.69E-40	-1.774	Down
STMN1	8.74E-24	1.93	Up	ATP8B1	3.09E-22	-1.135	Down
TWIST1	1.26E-30	2.158	Up	AXL	1.43E-37	-1.875	Down
TNFSF9	1.40E-28	1.145	Up	BANK1	2.63E-07	-1.193	Down
GDF15	3.86E-07	1.021	Up	BCL6	2.79E-47	-2.579	Down
IDO1	2.88E-36	1.974	Up	BEST1	4.34E-21	-1.187	Down
MDM2	7.42E-15	1.081	Up	BTK	2.10E-22	-1.268	Down
SEMA7A	7.04E-16	1.739	Up	C1QTNF1	3.59E-16	-1.269	Down
IGFBP3	1.71E-49	3.522	Up	C3	1.06E-05	-1.419	Down
KIT	1.13E-21	1.017	Up	C3AR1	2.04E-11	-1.533	Down
STC1	1.10E-31	2.334	Up	C5AR1	5.08E-17	-1.437	Down
LY6E	1.11E-14	1.594	Up	CAT	2.27E-23	-1.697	Down
HMGB2	2.01E-22	1.301	Up	CCL13	4.39E-14	-1.775	Down
HPGD	4.63E-05	1.526	Up	CCL14	5.49E-11	-1.374	Down
ESM1	1.07E-49	3.158	Up	CCL2	5.74E-36	-3.263	Down
TMEM100	1.35E-10	1.083	Up	CCL21	3.01E-19	-3.415	Down
IGF2	1.08E-41	6.824	Up	CCL23	1.17E-37	-2.356	Down
PBK	6.38E-26	2.327	Up	CCR1	4.11E-18	-1.418	Down
HSPA4	7.78E-19	1.161	Up	CD14	2.97E-15	-2.528	Down
SOCS1	3.34E-24	1.835	Up	CD163	1.49E-35	-3.429	Down
TNFRSF4	7.00E-47	1.379	Up	CD209	6.37E-14	-1.36	Down
ISG15	8.36E-19	1.447	Up	CD300A	8.51E-09	-1.005	Down
FANCA	2.90E-20	1.12	Up	CD38	1.18E-27	-1.399	Down
ZP3	7.64E-19	1.33	Up	CD4	2.74E-07	-1.078	Down
HSH2D	3.56E-18	1.063	Up	CD55	2.85E-15	-1.437	Down
MARCKS	1.26E-13	1.001	Up	CD68	2.90E-06	-1.648	Down
				CD84	4.13E-23	-1.117	Down
MYLK	4.28E-27	-1.711	Down	CEACAM1	5.61E-10	-1.172	Down
PDE4A	2.11E-32	-1.532	Down	CEBPD	6.08E-18	-1.661	Down

SPI1	5.56E-14	-1.383	Down	CELA1	3.08E-11	-1.082	Down
ROCK1	2.82E-31	-1.211	Down	CELF1	2.96E-21	-1.051	Down
HDAC4	1.08E-37	-1.264	Down	CFB	3.03E-13	-2.423	Down
IFI35	1.95E-16	-1.195	Down	CFH	1.95E-31	-2.391	Down
TTC7A	3.12E-25	-1.258	Down	CFLAR	1.58E-23	-1.058	Down
IP6K2	2.52E-18	-1.099	Down	CHGA	3.64E-12	-2.332	Down
RORA	2.07E-20	-1.582	Down	CHI3L1	2.56E-08	-1.017	Down
FSTL3	1.64E-14	-1.399	Down	CIITA	2.30E-20	-1.207	Down
VASH1	2.70E-16	-1.296	Down	CITED2	1.56E-13	-1.644	Down
SREBF1	4.69E-37	-2.761	Down	CKM	1.85E-22	-1.302	Down
FCGR2B	2.72E-09	-1.254	Down	CLDN1	2.76E-07	-1.382	Down
IL4R	9.13E-44	-2.62	Down	CLEC5A	4.81E-17	-1.109	Down
LXN	1.01E-23	-1.894	Down	CLEC7A	1.86E-29	-2.174	Down
PTPRC	1.55E-20	-1.447	Down	CMKLR1	5.08E-32	-1.718	Down
PHLPP1	1.55E-14	-1.169	Down	CNR1	4.95E-11	-1.578	Down
GCKR	1.63E-52	-2.714	Down	CNTN1	2.84E-11	-1.275	Down
MMP2	1.49E-40	-3.01	Down	COL1A1	0.00018	-1.08	Down
SIGLEC1	3.47E-31	-2.519	Down	CP	1.04E-07	-1.21	Down
SLC23A2	4.02E-12	-1.115	Down	CPT1A	3.48E-22	-1.269	Down
ICAM1	1.01E-09	-1.277	Down	CRHBP	3.63E-51	-2.945	Down
IRAK3	8.07E-47	-1.513	Down	CRLF1	9.94E-17	-1.705	Down
LYZ	0.000308	-1.178	Down	CRYAB	3.59E-20	-1.866	Down
THPO	7.55E-22	-1.295	Down	CSF1	1.87E-31	-2.547	Down
UNC13D	1.35E-27	-1.636	Down	CSF1R	1.36E-14	-1.856	Down
GGT5	4.39E-17	-1.856	Down	CSF3R	4.11E-31	-2.012	Down
MAPK1	1.27E-20	-1.324	Down	CTSK	4.46E-15	-1.209	Down
TIMP3	5.87E-10	-1.435	Down	CXCL12	8.99E-34	-3.008	Down
RHBDD3	5.45E-23	-1.137	Down	CXCL2	1.76E-13	-1.65	Down
MTMR3	3.19E-43	-1.809	Down	CYBA	2.53E-09	-1.336	Down
EP300	4.28E-24	-1.212	Down	CYBB	1.12E-18	-2.069	Down
SRSF5	2.37E-18	-1.206	Down	CYP11B2	1.74E-20	-3.851	Down
PROCR	3.05E-12	-1.123	Down	CYP26B1	1.98E-05	-1.022	Down
SLC17A9	8.65E-41	-1.934	Down	CYP2C8	1.98E-23	-1.255	Down
HCK	5.53E-17	-1.418	Down	CYSLTR2	0.008	-1.261	Down
KLF5	7.43E-24	-1.206	Down	DAPK1	3.56E-09	-1.185	Down
PARP4	5.33E-12	-1.03	Down	DBH	5.01E-14	-1.165	Down
NFAT5	2.58E-25	-1.437	Down	DCN	8.01E-37	-4.816	Down
SETD6	1.33E-23	-1.023	Down	DDX3Y	3.89E-07	-4.86	Down
WFDC1	1.32E-45	-2.645	Down	DDX5	8.55E-21	-1.349	Down
ZDHHC2	3.26E-13	-1.224	Down	DNASE1L3	2.64E-34	-2.926	Down
IKBKB	3.67E-28	-1.412	Down	DOCK2	1.74E-13	-1.066	Down
PLAT	1.08E-25	-2.547	Down	DUOX1	1.06E-65	-3.776	Down
NDRG1	1.05E-08	-1.266	Down	DUOXA1	3.63E-53	-2.128	Down
GSDMD	1.84E-13	-1.353	Down	ECM1	2.05E-05	-1.037	Down

LILRB1	1.02E-26	-1.414	Down	EDIL3	1.90E-08	-1.641	Down
TGFB1	2.61E-10	-1.088	Down	EDNRB	2.87E-13	-1.273	Down
SCN1B	7.75E-19	-1.805	Down	EGFR	2.95E-11	-1.146	Down
RARRES2	4.16E-15	-1.724	Down	EGR1	6.20E-07	-1.067	Down
PTGR1	1.85E-11	-1.232	Down	ELN	4.92E-19	-2.093	Down
PTGDS	2.00E-41	-6.285	Down	ENG	1.08E-21	-1.251	Down
GATA3	1.50E-06	-1.16	Down	FGR	4.69E-35	-1.671	Down
MAP3K8	1.34E-37	-2.35	Down	SLC7A2	6.08E-15	-1.539	Down
VTN	2.12E-08	-1.488	Down	SARM1	1.90E-22	-1.205	Down
SOD3	3.46E-21	-1.622	Down	PON1	8.72E-17	-2.387	Down
IL10RA	1.01E-27	-1.888	Down	ITGA3	1.61E-09	-1.105	Down
TCIRG1	2.00E-27	-2.179	Down	TAC1	6.50E-24	-1.77	Down
KRT18	0.0223	-1.434	Down	STAB1	9.19E-33	-2.657	Down
SCNN1A	2.52E-42	-3.07	Down	FMO1	1.09E-14	-2.037	Down
LPCAT3	8.98E-14	-1.045	Down	WAS	4.95E-19	-1.306	Down
SOD2	1.85E-14	-1.52	Down	IGF1	1.51E-21	-1.512	Down
PDGFRB	7.84E-13	-1.264	Down	SLC11A1	1.28E-59	-3.723	Down
OGG1	1.92E-22	-1.051	Down	MARCO	6.62E-29	-3.537	Down
FGF12	3.07E-18	-1.711	Down	HGF	1.80E-29	-2.475	Down
HYAL1	3.70E-07	-1.219	Down	GCLM	1.63E-24	-1.608	Down
VIPR1	1.82E-39	-1.448	Down	NR1H3	1.25E-35	-2.304	Down
POMC	4.80E-12	-2.223	Down	TYMP	3.71E-19	-2.043	Down
FNDC4	3.07E-23	-1.684	Down	FAS	2.26E-09	-1.035	Down
REG1A	6.40E-19	-2.239	Down	TIMP2	4.37E-09	-1.137	Down
IGFBP5	7.06E-46	-3.358	Down	FLT4	3.16E-30	-1.427	Down
IL1R1	9.80E-14	-1.294	Down	VCAN	4.31E-16	-2.391	Down
IL1RL1	2.96E-51	-3.921	Down	MSR1	9.54E-19	-1.523	Down
IL18R1	5.34E-19	-1.122	Down	LCP2	6.90E-22	-1.416	Down
KYNU	1.99E-18	-1.498	Down	NEDD4L	1.12E-18	-1.26	Down
EPAS1	1.94E-14	-1.108	Down	PTGER3	0.00691	-1.043	Down
PLA2G4A	7.59E-39	-2.368	Down	ITIH4	3.12E-42	-3.459	Down
F3	1.30E-13	-1.36	Down	TRAF1	3.56E-22	-1.141	Down
HSD11B1	8.38E-26	-2.125	Down	TBXAS1	2.58E-16	-1.297	Down
IRF6	5.93E-09	-1.772	Down	NGFR	6.68E-35	-2.309	Down
TNNT2	4.37E-34	-1.727	Down	IL33	2.44E-36	-1.812	Down
NRP2	5.15E-22	-1.848	Down	IER3	7.60E-22	-2.075	Down
SGK1	5.87E-18	-1.632	Down	TLR2	1.38E-18	-1.14	Down
PKD2	1.20E-23	-1.67	Down	IL18BP	3.45E-20	-1.241	Down
TGFB3	3.42E-26	-1.527	Down	YAP1	1.16E-19	-1.361	Down
TEK	1.50E-46	-1.873	Down	THBS1	4.43E-43	-3.385	Down
KCNJ5	2.80E-30	-4.269	Down	MAPKBP1	2.04E-47	-1.637	Down
TGFBI	0.00296	-1.134	Down	RBP4	3.84E-13	-2.601	Down
PTK2B	1.58E-27	-1.345	Down	IDH1	9.22E-12	-1.309	Down
TNFSF10	3.43E-07	-1.674	Down	SLC40A1	2.07E-19	-3.987	Down

PTGFR	0.000102	-1.103	Down	PARP9	6.34E-16	-1.097	Down
MMP19	1.68E-35	-1.905	Down	KIAA1109	7.81E-25	-1.318	Down
LRP1	2.47E-19	-1.608	Down	LUM	6.52E-33	-4.085	Down
PLP1	2.91E-05	-1.088	Down	MAP3K12	5.56E-16	-1.184	Down
PTGIS	1.62E-42	-1.529	Down	ESR2	1.29E-32	-1.672	Down
SNAI1	3.42E-31	-1.9	Down	FBLN5	7.78E-30	-2.309	Down
HIF3A	8.96E-19	-2.619	Down	FGF7	4.37E-20	-1.172	Down
F13A1	1.07E-25	-3.149	Down	TPM1	1.34E-20	-1.222	Down
STAT5A	7.26E-37	-2.068	Down	NTRK3	6.74E-37	-1.137	Down
KLF2	4.04E-20	-1.623	Down	IRF8	1.78E-29	-1.463	Down
IRF5	4.67E-18	-1.261	Down	KSR1	8.44E-34	-1.768	Down
PLD2	9.63E-34	-1.446	Down	PIK3R5	2.10E-42	-1.85	Down
SIGLEC9	1.82E-16	-1.566	Down	SMAD4	2.95E-23	-1.447	Down
RIPK3	1.56E-43	-1.314	Down	ERBB2	1.56E-13	-1.03	Down
RNASE1	0.00452	-1.158	Down	IGFBP4	1.44E-25	-2.567	Down
RHBDF2	8.25E-21	-1.324	Down	SIGLEC10	1.45E-36	-2.469	Down
THEMIS2	1.04E-09	-1.332	Down	SLC2A5	8.15E-10	-1.392	Down
LILRB2	6.21E-20	-1.294	Down	EPHA2	4.55E-28	-1.551	Down
NR0B2	0.0248	-1.201	Down	FCGR2A	5.32E-19	-1.568	Down
PTPRE	1.02E-14	-1.238	Down	RORC	0.000561	-1.332	Down
SERPINF1	5.11E-14	-1.577	Down	S100A8	1.96E-31	-3.39	Down
LYVE1	2.01E-10	-1.778	Down	NFKBIZ	7.41E-36	-1.998	Down
GSTM1	0.0104	-4.763	Down	SLIT2	6.48E-19	-1.999	Down
SAA2	7.04E-28	-2.908	Down	SFRP2	0.00112	-1.396	Down
PDGFRA	2.41E-47	-3.068	Down	OSMR	3.17E-20	-1.402	Down
HAVCR2	1.09E-10	-1.02	Down	GPC3	3.36E-14	-1.451	Down
GLUL	8.79E-24	-2.047	Down	STAR	2.45E-07	-1.2	Down
IL10	1.59E-19	-1.009	Down	PLIN2	1.40E-29	-2.639	Down
TLR4	7.49E-39	-1.737	Down	SERPING1	2.23E-29	-2.574	Down
MYC	7.61E-21	-1.759	Down	NCAM1	5.87E-08	-1.017	Down
PLCB3	9.77E-23	-1.153	Down	S100B	2.24E-22	-1.861	Down
IL18	6.94E-07	-1.244	Down	FCN2	2.52E-14	-2.184	Down
NEK7	2.43E-19	-1.363	Down	S100A1	2.70E-14	-1.096	Down
GJA1	6.69E-16	-1.559	Down	NLRX1	3.98E-14	-1.021	Down
MERTK	3.45E-10	-1.125	Down	TNFSF13	3.25E-11	-1.069	Down
NR4A2	9.27E-12	-1.527	Down	PDZK1IP1	0.000483	-1.616	Down
MCOLN2	1.06E-31	-2.369	Down	PDPN	4.58E-08	-1.389	Down
PRKCA	1.94E-12	-1.327	Down	NFIA	1.59E-23	-1.201	Down
MAPK13	4.26E-25	-1.762	Down	HAAO	4.67E-27	-1.724	Down
TIMP4	8.40E-29	-2.171	Down	S100A9	1.41E-33	-3.895	Down
IL34	5.15E-07	-1.689	Down	FZD5	2.27E-21	-1.045	Down
TNFRSF14	6.16E-22	-1.71	Down	FSTL1	2.98E-12	-1.391	Down
SLAMF8	7.00E-12	-1.178	Down	TGFBR2	4.06E-18	-1.373	Down
SCUBE1	1.04E-05	-1.041	Down	NFASC	2.87E-22	-2.045	Down

ZYX	1.67E-12	-1.033	Down	PTX3	1.72E-11	-1.593	Down
PTGIR	1.25E-35	-1.774	Down	ZC3H12A	7.25E-27	-1.532	Down
FNDC5	2.04E-32	-2.196	Down	NPY5R	1.32E-49	-1.502	Down
TFF3	2.08E-21	-1.673	Down	ITGA2	9.37E-11	-1.287	Down
CBS	0.000794	-1.169	Down	SHH	0.000114	-1.088	Down
ITGB2	1.68E-06	-1.203	Down	TNFRSF11	1.37E-22	-1.587	Down
GPER1	5.85E-07	-1.484	Down	MUC1	1.62E-17	-1.748	Down
FREM1	9.99E-24	-1.515	Down	IRF7	4.84E-10	-1.056	Down
LRFN5	2.55E-27	-1.124	Down	NR2F2	9.83E-17	-1.236	Down
FOLR2	8.17E-05	-1.185	Down	RXRA	3.12E-21	-1.304	Down
PBX3	2.81E-14	-1.229	Down	PDE2A	0.00553	-1.117	Down
SPINT2	1.78E-11	-1.17	Down	LILRB4	2.64E-10	-1.05	Down
KLK1	0.0211	-1.219	Down	PPARA	2.56E-21	-1.028	Down
KRT1	4.70E-22	-1.067	Down	MITF	3.76E-16	-1.07	Down
MLKL	9.87E-28	-1.258	Down	FPR3	1.21E-15	-1.293	Down
STAT3	9.24E-31	-1.491	Down	SEMA4D	3.05E-26	-1.295	Down
RNASE2	1.36E-07	-1.478	Down	PEAR1	4.72E-62	-2.209	Down
GKN1	1.41E-31	-1.118	Down	PLA2G2A	3.85E-19	-2.551	Down
ITGAM	4.60E-35	-2.011	Down	S100A4	3.62E-08	-1.639	Down
FOS	3.10E-09	-1.393	Down	TCF4	4.83E-26	-1.644	Down
KRT8	1.02E-06	-1.43	Down	MAFG	5.89E-20	-1.045	Down
SERPINB9	9.21E-26	-1.736	Down	SERPINA1	2.05E-06	-1.412	Down
STAT2	3.61E-25	-1.299	Down	TRIM33	1.66E-33	-1.061	Down
FPR1	2.47E-23	-1.925	Down	MAP3K5	8.61E-40	-2.769	Down
PTGER4	4.02E-37	-1.714	Down	S100A10	5.66E-07	-1.162	Down
FGG	0.0112	-1.972	Down	LPAR1	5.07E-19	-1.825	Down
PIK3CD	3.67E-25	-1.181	Down	TGM2	1.23E-09	-1.253	Down
REG3A	6.92E-08	-1.419	Down	FCGR3A	2.70E-14	-1.865	Down
REG1B	5.41E-17	-1.192	Down	HSD3B2	6.98E-31	-5.328	Down
IL16	2.15E-16	-1.011	Down	LILRB3	1.03E-48	-2.374	Down
HSPA6	6.50E-08	-1.05	Down	GABBR1	7.01E-47	-2.219	Down
PARP14	1.40E-27	-1.147	Down	GPSM3	1.94E-13	-1.186	Down
SAA1	5.15E-33	-4.501	Down	LTB4R	2.77E-11	-1.036	Down
MST1	5.34E-25	-1.809	Down	LTB4R2	2.40E-31	-1.441	Down
STAT5B	4.51E-37	-1.792	Down	TNFRSF25	7.71E-40	-2.025	Down
RNF213	4.48E-32	-1.778	Down	ORM2	0.000133	-1.322	Down
SELP	1.11E-28	-1.352	Down	ORM1	1.78E-07	-2.076	Down
NPPA	1.84E-20	-1.107	Down	TNFSF12	6.15E-30	-1.761	Down
FOSL1	1.90E-06	-1.057	Down	LILRA2	2.27E-13	-1.022	Down
METRNL	3.47E-08	-1.189	Down	PLA2G4B	2.17E-25	-1.112	Down
MTHFR	1.08E-28	-1.236	Down	LYN	3.13E-11	-1.091	Down
NR2C2	5.30E-28	-1.185	Down	HP	2.72E-28	-2.639	Down
THBD	6.91E-16	-1.273	Down	MRC1	1.92E-30	-2.416	Down
SSTR2	7.79E-28	-1.033	Down	TRPV1	2.51E-28	-1.108	Down

RGMA	2.96E-29	-1.293	Down	SPON1	1.13E-59	-3.042	Down
SATB1	4.71E-25	-1.457	Down	IKBKE	3.98E-19	-1.054	Down
SLIT3	9.75E-12	-1.298	Down	TXNIP	2.49E-13	-1.942	Down
SOCS3	3.57E-11	-1.594	Down	RASSF5	2.09E-09	-1.007	Down
TNFAIP2	4.95E-25	-2.27	Down	ADORA3	2.09E-16	-1.392	Down
MUC1	1.62E-17	-1.748	Down				

Table S4. A total of 113 differentially expressed inflammation-related genes were associated with survival according to univariate regression analysis.

Genes	beta	P value	Hazard Ratio (95% CI)	Genes	beta	P value	Hazard Ratio (95% CI)
CYP26B1	0.197	0.000	1.22 (1.1-1.35)	ADGRE2	0.191	0.010	1.21 (1.04-1.4)
DCN	0.192	0.001	1.21 (1.08-1.35)	PLD2	0.411	0.008	1.51 (1.11-2.04)
CNTN1	0.166	0.000	1.18 (1.09-1.28)	AOC3	0.167	0.007	1.18 (1.05-1.33)
NR1H3	-0.234	0.047	0.79 (0.63-1)	SERPINF1	0.179	0.010	1.2 (1.04-1.37)
TIMP2	0.269	0.000	1.31 (1.14-1.5)	PDGFRA	0.209	0.004	1.23 (1.07-1.42)
VCAN	0.172	0.003	1.19 (1.06-1.33)	IL10	0.189	0.003	1.21 (1.06-1.38)
MSR1	0.132	0.038	1.14 (1.01-1.29)	CTSV	0.135	0.004	1.14 (1.04-1.25)
ELN	0.197	0.000	1.22 (1.09-1.36)	MYC	0.201	0.004	1.22 (1.06-1.41)
PTGER3	0.176	0.000	1.19 (1.08-1.31)	YAP1	0.316	0.004	1.37 (1.12-1.68)
ITIH4	-0.276	0.002	0.76 (0.64-0.9)	THBS1	0.240	0.001	1.27 (1.11-1.46)
NGFR	0.097	0.028	1.1 (1.01-1.2)	MAPKBP1	0.389	0.004	1.48 (1.13-1.92)
MYLK	0.205	0.000	1.23 (1.1-1.37)	CYP2C8	-0.145	0.008	0.87 (0.78-0.96)
HDAC4	0.309	0.012	1.36 (1.07-1.73)	DUSP5	0.159	0.033	1.17 (1.01-1.36)
ATP11A	0.227	0.009	1.25 (1.05-1.49)	LUM	0.232	0.000	1.26 (1.11-1.43)
TTC7A	0.348	0.004	1.42 (1.12-1.79)	FBLN5	0.152	0.042	1.16 (1-1.35)
FSTL3	0.254	0.000	1.29 (1.13-1.47)	FGF7	0.136	0.002	1.15 (1.05-1.25)
SREBF1	0.260	0.009	1.3 (1.07-1.58)	TPM1	0.231	0.004	1.26 (1.08-1.46)
FGFR1	0.194	0.003	1.21 (1.07-1.38)	KSR1	0.211	0.021	1.23 (1.03-1.48)
MMP2	0.161	0.016	1.17 (1.03-1.34)	EPHA2	0.286	0.001	1.33 (1.12-1.58)
HCN2	0.216	0.012	1.24 (1.05-1.46)	ECM1	0.258	0.000	1.29 (1.14-1.47)
MMP11	0.152	0.001	1.16 (1.06-1.27)	CTSK	0.174	0.008	1.19 (1.05-1.35)
MAPK1	0.347	0.020	1.41 (1.05-1.9)	S100A8	0.069	0.022	1.07 (1.01-1.14)
TIMP3	0.187	0.004	1.21 (1.06-1.37)	ACKR3	0.266	0.000	1.3 (1.13-1.5)
EP300	0.319	0.020	1.38 (1.05-1.81)	AGTR1	0.128	0.007	1.14 (1.04-1.25)
WFDC1	0.158	0.034	1.17 (1.01-1.36)	SLIT2	0.130	0.010	1.14 (1.03-1.26)
NDRG1	0.165	0.027	1.18 (1.02-1.37)	SFRP2	0.096	0.001	1.1 (1.04-1.17)
TGFB1	0.211	0.036	1.23 (1.01-1.51)	OSMR	0.137	0.026	1.15 (1.02-1.3)
CAV1	0.244	0.000	1.28 (1.13-1.44)	EGFR	0.253	0.000	1.29 (1.15-1.44)
PTGDS	0.119	0.012	1.13 (1.03-1.24)	NCAM1	0.140	0.001	1.15 (1.06-1.25)
CXCL12	0.123	0.029	1.13 (1.01-1.26)	ADAM33	0.141	0.011	1.15 (1.03-1.28)
COL1A1	0.205	0.000	1.23 (1.1-1.38)	ADAMTS12	0.210	0.000	1.23 (1.1-1.39)

SOD3	0.168	0.006	1.18 (1.05-1.33)	GJA1	0.163	0.019	1.18 (1.03-1.35)
CRYAB	0.212	0.000	1.24 (1.1-1.38)	ADAMTS1	0.134	0.045	1.14 (1-1.3)
VWF	0.221	0.031	1.25 (1.02-1.53)	IL34	0.173	0.033	1.19 (1.02-1.39)
SPARC	0.265	0.001	1.3 (1.11-1.53)	TNFRSF14	-0.239	0.044	0.79 (0.63-0.99)
PDGFRB	0.283	0.000	1.33 (1.13-1.56)	STC1	0.175	0.013	1.19 (1.04-1.36)
FNDC4	0.161	0.026	1.17 (1.02-1.35)	ZYX	0.284	0.030	1.33 (1.03-1.72)
IGFBP5	0.128	0.015	1.14 (1.02-1.26)	FNDC5	0.166	0.023	1.18 (1.02-1.36)
IL1R1	0.328	0.000	1.39 (1.15-1.67)	PDPN	0.173	0.004	1.19 (1.06-1.34)
EPAS1	0.265	0.017	1.3 (1.04-1.63)	NFIA	0.211	0.034	1.24 (1.02-1.5)
SLC2A1	0.194	0.012	1.21 (1.04-1.41)	FSTL1	0.302	0.001	1.35 (1.14-1.61)
F3	0.140	0.005	1.15 (1.04-1.27)	NFASC	0.192	0.005	1.21 (1.06-1.38)
NRP2	0.220	0.001	1.25 (1.09-1.42)	EDIL3	0.240	0.001	1.27 (1.11-1.46)
TGFB3	0.207	0.005	1.23 (1.06-1.42)	LRFN5	0.149	0.002	1.16 (1.06-1.27)
TGFBI	0.138	0.014	1.15 (1.03-1.28)	TMEM100	0.116	0.026	1.12 (1.01-1.24)
EGR1	0.221	0.001	1.25 (1.1-1.42)	IGF2	0.075	0.013	1.08 (1.02-1.14)
ADCY7	0.254	0.000	1.29 (1.12-1.48)	AXL	0.171	0.011	1.19 (1.04-1.35)
PTGFR	0.156	0.001	1.17 (1.06-1.29)	KRT1	0.086	0.002	1.09 (1.03-1.15)
TWIST1	0.152	0.005	1.16 (1.04-1.3)	STAT3	0.282	0.042	1.33 (1.01-1.74)
MMP19	0.189	0.021	1.21 (1.03-1.42)	FOS	0.171	0.009	1.19 (1.04-1.35)
LRP1	0.385	0.000	1.47 (1.21-1.78)	C1QTNF1	0.144	0.029	1.15 (1.02-1.31)
PTGIS	0.155	0.000	1.17 (1.08-1.26)	FOSL1	0.166	0.011	1.18 (1.04-1.35)
SNAI1	0.176	0.020	1.19 (1.03-1.38)	RGMA	0.158	0.001	1.17 (1.06-1.29)
F13A1	0.115	0.040	1.12 (1.01-1.25)	TCF4	0.235	0.011	1.27 (1.06-1.52)
TNFSF9	0.130	0.016	1.14 (1.03-1.26)	HSH2D	-0.144	0.055	0.87 (0.75-1)
C3	0.114	0.027	1.12 (1.01-1.24)	MAFG	0.297	0.017	1.35 (1.06-1.71)
TGM2	0.165	0.002	1.18 (1.06-1.31)				

Table S5. The primer sequences of six IRGs for qPCR assay.

Primer name	Forward sequence (5' -> 3')	Reverse sequence (5' -> 3')
TNFRSF12A	GATCCAGTGACAATGTGCC	AGACTGGCTCTAGAATGGA
NR1H3	TCTGGAGACATCTCGGAGGTA	GGCCCTGGAGAACTCGAAG
ITIH4	TCCGGTTCAAGCCAACACTTT	CATAGCGGATAATGAGGTTGCC
IL1R1	GGCTGAAAAGCATAGAGGGAAC	CTGGGCTCACAATCACAGG
ELN	ACATCTACAAGTGTTCAGGCATC	TGGTTACCAAACTGGTGGTCA
CYP26B1	GGCAACGTGTTCAAGACGC	TGCTCGCCCATGAGGATCT
GAPDH	TGGTGAAGACGCCAGTGGA	GCACCGTCAAGGCTGAGAAC
